# Molecular characterization of firefly nuptial gifts: a multi-omics approach sheds light on postcopulatory sexual selection

**DOI:** 10.1038/srep38556

**Published:** 2016-12-22

**Authors:** Nooria Al-Wathiqui, Timothy R. Fallon, Adam South, Jing-Ke Weng, Sara M. Lewis

**Affiliations:** 1 Department of Biology, Tufts University, Medford, MA, 02155, USA; 2 Whitehead Institute for Biomedical Research, 9 Cambridge Center, Cambridge, MA, 02142, USA; 3 Department of Biology, Massachusetts Institute of Technology, Cambridge, MA, 02139, USA; 4 Department of Immunology and Infectious Disease, Harvard T.H. Chan School of Public Health, Boston, MA, 02115, USA

## Abstract

Postcopulatory sexual selection is recognized as a key driver of reproductive trait evolution, including the machinery required to produce endogenous nuptial gifts. Despite the importance of such gifts, the molecular composition of the non-gametic components of male ejaculates and their interactions with female reproductive tracts remain poorly understood. During mating, male *Photinus* fireflies transfer to females a spermatophore gift manufactured by multiple reproductive glands. Here we combined transcriptomics of both male and female reproductive glands with proteomics and metabolomics to better understand the synthesis, composition and fate of the spermatophore in the common Eastern firefly, *Photinus pyralis*. Our transcriptome of male glands revealed up-regulation of proteases that may enhance male fertilization success and activate female immune response. Using bottom-up proteomics we identified 208 functionally annotated proteins that males transfer to the female in their spermatophore. Targeted metabolomic analysis also provided the first evidence that *Photinus* nuptial gifts contain lucibufagin, a firefly defensive toxin. The reproductive tracts of female fireflies showed increased gene expression for several proteases that may be involved in egg production. This study offers new insights into the molecular composition of male spermatophores, and extends our understanding of how nuptial gifts may mediate postcopulatory interactions between the sexes.

A powerful driver of evolutionary change, sexual selection is responsible for shaping myriad traits that impact reproductive success for individuals of each sex. It is now widely recognized that both intrasexual competition and intersexual choice continue to operate beyond the end of copulation^1^,^2^. These prolonged sexual interactions play out inside female reproductive tracts, and are accomplished through the action of diverse molecules manufactured by both sexes^3^. Polyandry results in temporal and spatial overlap of male ejaculates, and thus heightens selective pressure on males to maximize their own paternity success relative to that of their rivals^1^,^4^,^5^. One potential result is the elaboration of male reproductive glands that manufacture substances to increase relative reproductive success^1^,^4^. During copulation, males in diverse species deliver to females not only sperm, but a complex array of seminal products including proteins, hormones, nucleic acids, lipids, amino acids, carbohydrates and defensive compounds^6^,^7^. Known as endogenous nuptial gifts, these non-gametic components of male ejaculates can be transferred either via seminal fluid or within a sperm-containing package known as a spermatophore^8^,^9^. Among gift components, seminal fluid proteins (SFPs) have been characterized most extensively, particularly in humans and the fruit fly *Drosophila melanogaster*^10^. Male *D. melanogaster* transfer more than 200 distinct SFPs to their mates during copulation, and human seminal fluid contains approximately 100 different SFPs^10^,^11^,^12^,^13^,^14^. Despite the remarkable diversity and rapid evolution of SFPs^15^,^16^, the major protein classes are highly conserved, suggesting functional similarities across taxa even as distant as insects and mammals^17^,^18^.

Interestingly, SFPs have been shown to dramatically alter female physiology and behavior once deposited inside the female reproductive tract. For instance, in *D. melanogaster*, SFPs reduce female receptivity to further mating, increase oogenesis and oviposition, alter female sperm storage and use, and change female feeding and sleep patterns^3^,^10^,^19^. However, despite decades of research on nuptial gifts in select taxa, the detailed molecular mechanisms underlying how such gifts influence postcopulatory sexual selection remain largely unresolved. Transcriptomic studies of the male accessory glands (MAGs) that are responsible for manufacturing SFPs have been restricted primarily to *Drosophila* and other dipterans^20^,^21^,^22^. Although detailed anatomical descriptions of MAGs do exist for other taxa^23^, their glandular products remain poorly characterized. Additionally, sexual selection research shows a recognized bias toward male reproductive traits^24^. Thus, despite the central role that females play in postcopulatory sexual interactions, remarkably little is known about the products of female reproductive glands^25^,^26^,^27^,^28^,^29^,^30^. Understanding the role of nuptial gifts in the context of sexual selection will require comprehensive analyses interrogating the molecular composition of male nuptial gifts as well as secretions from female reproductive tissues that receive and process male gifts.

Fireflies are bioluminescent beetles belonging to the family Lampyridae, which comprises ~2000 extant species^31^. Their diverse life histories, sexual signals, and mating systems have made fireflies an important group for understanding the evolution of nuptial gifts^32^,^33^,^34^,^35^. The firefly *Photinus pyralis* is a common North American species widely distributed across the eastern United States^36^. Historically important, *P. pyralis* was used for early studies focused on the biochemistry and physiology of bioluminescence^36^,^37^,^38^, as well as precopulatory sexual selection^39^. Within the genus *Photinus*, males deliver nuptial gifts in the form of elaborate spermatophores that are manufactured by multiple reproductive accessory glands^34^,^40^. Because most *Photinus* fireflies do not eat in their adult stage, all reproductive activities must be fueled by stored resources acquired during larval feeding^41^. This is reflected in the decline of spermatophore size over successive matings^42^. Although the production of nuptial gifts is costly for males, larger gifts are correlated with increased reproductive success^43^. Male gifts also provide multiple benefits to females. While *Photinus* females are polyandrous, capable of mating with multiple males over successive nights, females mate with only a single male per night. Compared to females that mated only once, triply-mated *Photinus* females showed 73% greater lifetime fecundity^44^. Furthermore, females that receive larger nuptial gifts showed a 12–16% increase in their longevity^43^,^44^. Radiolabeling studies have shown that some spermatophore-derived proteins become incorporated into the developing oocytes of *Photinus* females^45^. Thus, nuptial gifts have major fitness consequences for both male and female fireflies.

Nearly 25% of all firefly species exhibit extreme sexual dimorphism: adult females completely lack wing development or have greatly reduced wings and are thus incapable of flight^46^. Physiological tradeoffs between flight and reproduction are well documented in other insects^47^,^48^, with flightlessness shifting resource allocation toward increased reproductive output^47^,^48^,^49^,^50^,^51^,^52^. Recent phylogenetic analysis revealed that female flightlessness has evolved repeatedly in the Lampyridae, typically followed by loss of male nuptial gifts^35^. Such correlated evolution between male and female traits suggests that firefly nuptial gifts not only mediate postcopulatory sexual selection, but may also be intimately linked with patterns of female reproductive investment. Thus, better understanding of the molecular composition of firefly nuptial gifts may provide new insights into their role in postcopulatory sexual interactions as well as their influence on other key life history traits.

In *P. pyralis*, as in many other *Photinus* fireflies, the male spermatophore is produced by four distinctive paired reproductive accessory glands (Fig. 1a)^40^. Most prominent are the tightly coiled spiral accessory glands (SpAGs). Prior to mating, the lumen of each spiral gland contains a secretion edged with two longitudinal rows of serrated scales (Fig. 1b). During mating, the spiral glands empty (Fig. 1a), and their secretions fuse to form the major structural component of the spermatophore. As it passes through the male ejaculatory duct (Ej; Fig. 1a), this spermatophore acquires additional material, including sperm rings that have been stored in the seminal vesicles (Fig. 1b) and the contents of three additional pairs of tubular accessory glands: the short, medium, and long accessory glands, here termed other accessory glands (OAG) (Fig. 1b). Spermatophore transfer from the male to the female bursa copulatrix (B; Fig. 1a) takes about 30–60 min. Sperm rings are released into the female sperm storage organ, the spermatheca (Spt, Fig. 1a,c), then disperse as sperm become capacitated and begin swimming slowly in dense aggregations. The rest of the male spermatophore enters a specialized female structure known as the spermatophore-digesting gland (SDG; Fig. 1a and d), where it disintegrates within 2–3 days after copulation.

Here, we adopted a multi-omics approach to interrogate the synthesis, content and fate of the spermatophore nuptial gift in *P. pyralis*. We sequenced and analyzed the transcriptomes of both male and female reproductive tissues, which revealed unique patterns of gene expression in these tissues. We further carried out bottom-up MS/MS proteomics and liquid-chromatography high-resolution accurate-mass spectrometry (LC-HRAM-MS)-based metabolomics to explore the molecular composition of *P. pyralis* spermatophores at the protein and metabolite levels, respectively. Importantly, this work is part of an expanding set of non-model organisms that lack a sequenced genome yet have biologically interesting reproductive molecules that can be identified using a combination of *de novo* transcriptomes, proteomics and RNA sequencing. Indeed, similar approaches have identified reproductive molecules in crickets^53^, moths^25^, and butterflies^54^, expanding our knowledge of nuptial gifts beyond existing model systems such as *Drosophila* and humans, and further shedding light on specific accessory gland functions and the molecular mechanisms of postcopulatory sexual selection.

## Results

### Probing *P. pyralis reproductive tissue* gene expression profiles by RNAseq

To elucidate gene expression in specific male accessory glands that manufacture nuptial gifts as well as in the female tissues that receive and process such gifts, we used RNAseq to assemble a transcriptome of *P. pyralis* reproductive tissues. We successfully demultiplexed a total of 320,271,148 reads into 18 separate libraries, each containing an average of 17,792,841 sequences. All libraries were assembled into a *de novo* transcriptome containing 47,131 contigs with an average contig length of 1159 bp. Gene expression patterns indicated strong tissue-specificity, and biological coefficient of variation analysis based on normalized read counts demonstrated the expected clustering of biological replicates within each *P. pyralis* male tissue (Supplementary Fig. 1).

### Differential gene expression in male reproductive glands

**Figure 2:**
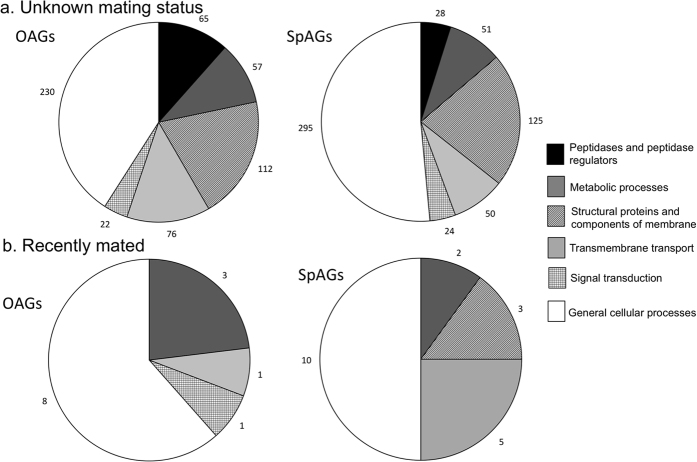
Distributions of gene ontology categories for *P. pyralis* genes up-regulated in males’ other accessory glands (OAGs) and spiral accessory glands (SpAGs), both compared to thorax for: (**a**) males whose mating status was unknown, and (**b**) males that had mated within the previous 2 h.

To examine *P. pyralis* differential gene expression in the spiral accessory glands and other accessory glands, we identified transcripts that showed a log_2_ fold change (logFC) ≥ 2 in these reproductive glands compared to male thorax, and also showed a false discovery rate (FDR) ≤ 0.01. Our transcriptome analysis identified 3294 putative transcripts that were significantly up-regulated in the major accessory glands compared to male thorax (Supplementary Table 1). Both types of male accessory glands showed similar gene ontology (GO) functional categories (Molecular Function, Level III), including peptidases and peptidase regulators, metabolic processes, structural proteins, transmembrane transport and signal transduction (Fig. 2a). In other male accessory glands, 11.5% of the transcripts had functions related to peptidase and peptidase regulators, compared to only 4.8% of genes in the spiral accessory glands (Fig. 2a).

To gain insight into differentiated function between these male glands, we first identified sequences that were significantly up-regulated with LogFC ≥ 10 in either male spiral accessory glands or other accessory glands compared to thorax, then identified sequences that were significantly differentially expressed between the two male gland types (LogFC ≥ 2; FDR ≤ 0.01). Comparison of GO functional categories for this subset of uniquely expressed genes confirmed that other accessory glands were mainly enriched in peptidase and peptidase regulator activities (Table 1).

**Figure 3:**
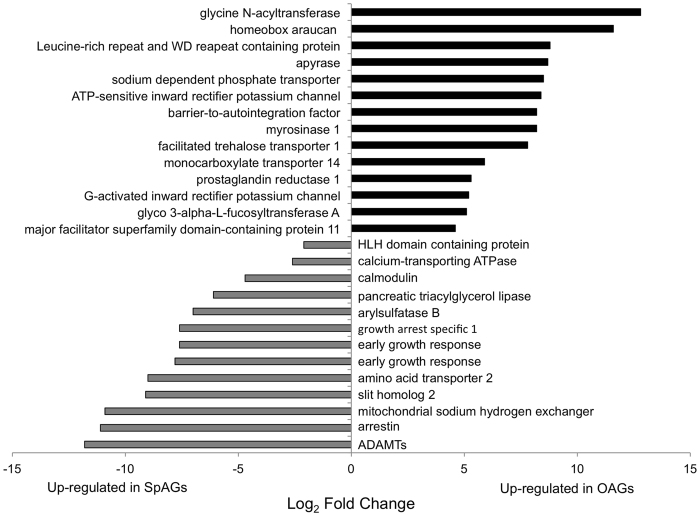
Comparison of differences in gene expression (log2 fold change) for annotated sequences co-expressed in other accessory glands (OAGs) and spiral accessory glands (SpAGs) of *P. pyralis* males.

We further characterized differences between male spiral and other accessory glands by comparing expression levels of sequences co-expressed in both tissues (Fig. 3; Supplementary Table 2). The 14 annotated genes that were up-regulated in males’ other accessory glands compared to spiral accessory glands were predicted to be involved in general cellular processes. The 13 genes up-regulated in male spiral accessory glands compared to other male accessory glands (Fig. 3) included a homolog to a metalloprotease, a disintegrin and metalloproteinase with a thrombospondin motif (ADAMTS; DN15036_c0_g1_i8).

### Effects of mating on male gene expression

We also examined reproductive gene expression in *P. pyralis* males 2 h after mating, a time when they are actively manufacturing new spermatophores. We identified 206 sequences in the spiral accessory glands and 253 sequences in the other accessory glands that were up-regulated in each tissue compared to thorax and contained a secretion signal (Supplementary Table 1). Of these, 402 were uniquely expressed in only one type of male accessory gland. In comparison to other males (Fig. 2a), the other accessory glands of recently mated males showed an increase in metabolic processes (Fig. 2b), particularly purine and cysteine metabolism, while the spiral accessory glands of recently mated males showed an increase in transmembrane transport function (Fig. 2b), primarily amino acid transporters.

### Protein composition of the firefly nuptial gift

**Figure 4:**
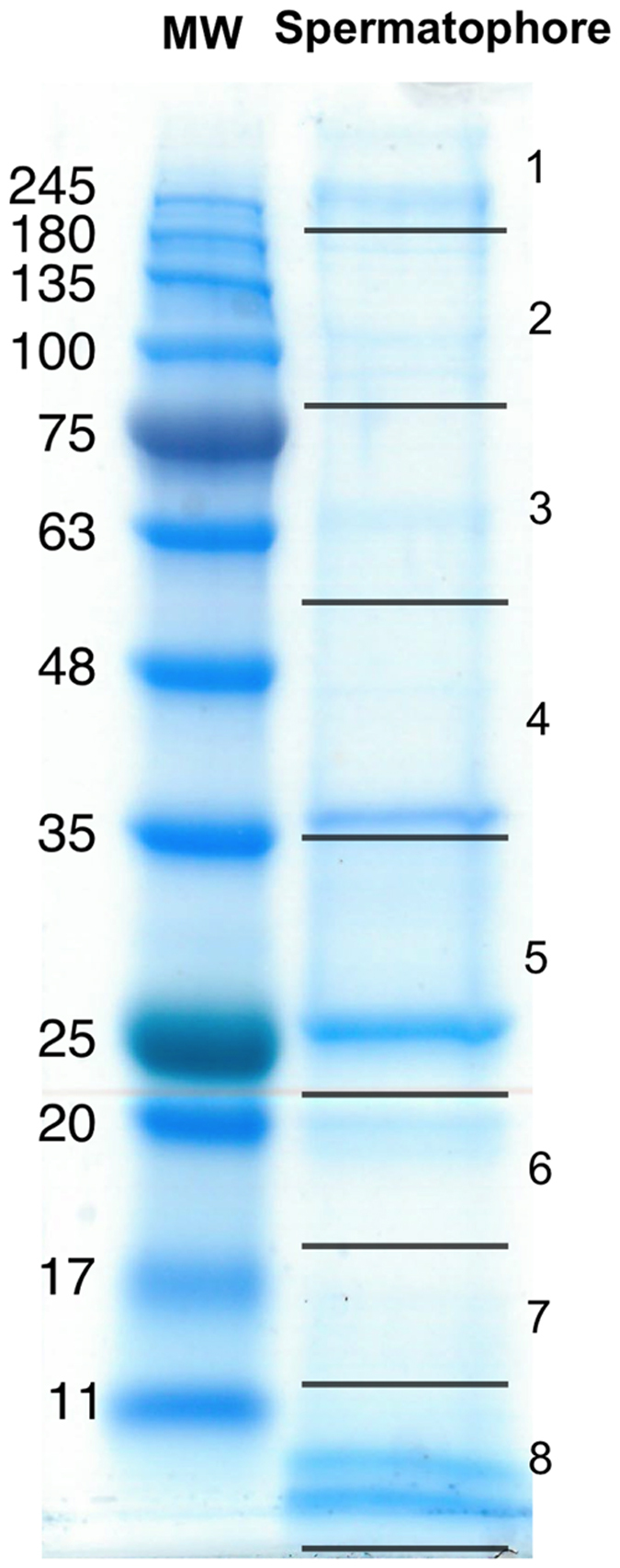
SDS-PAGE gel of soluble protein extract from a single *P. pyralis* male spermatophore with BLUEstain™ Protein MW ladder. Right-hand numbers indicate gel sections excised for proteomic analysis. Proteomic data from individual gel sections is available online (PRIDE Supplementary Data).

To examine the composition of *P. pyralis* nuptial gifts, we dissected a spermatophore from a mated female immediately after copulation, separated solubilized proteins on a SDS-PAGE gel (Fig. 4), and examined protein composition by digestion of proteins into peptides followed by nano LC-HRAM-MS/MS proteomic analysis. Combined with transcriptome data from *P. pyralis* male accessory glands and fat body, this approach allowed us to identify 425 proteins that were transferred to females in the male spermatophore. Of these, 208 were annotated by identifying homologs in other organisms using Blast2go and InterProScan (Supplementary Table 4). Based on our male transcriptome results, we were also able to determine the putative anatomical production site for 68 of these spermatophore proteins (Table 2; Supplementary Table 3). As the spermatophore is extracellular, proteins that are packaged into the spermatophore presumably must first be secreted, though this is not the only possible mechanism of spermatophore incorporation. To identify protein products detected in the spermatophore that may be secreted, we performed an in silico prediction of signal peptide sequences. This analysis revealed that many of the proteins identified via proteomics and that were associated with differentially expressed transcripts do indeed contain predicted signal peptides (Tables 2 and 3; Supplementary Table 3).

The spiral accessory glands were identified as the production site for two serine peptidases. One of these, a transcript with homology to the peptidase Snake (DN10938_c0_g1_i1), showed a LogFC of 9.8 compared to male thorax (Table 2; Supplementary Table 3), which is within the top 8% of differentially expressed genes in this male gland. Snake is a member of the protease cascade that leads to the activation of the Toll pathway, which is important for *Drosophila* embryonic development and immune response activation^55^. Another peptidase (DN8730_c0_g1_i1) showed homology to trypsin 1; with a LogFC of 8.5, this transcript is in the top 15% of most differentially expressed genes compared to male thorax (Table 2).

Proteomics also confirmed the presence in the *P. pyralis* spermatophore of several male reproductive proteins apparently manufactured by other male accessory glands (Table 2; Supplementary Table 3). Among the peptidases, one transcript (DN14826_c0_g1_i1; LogFC = 3.5 compared to male thorax) showed significant similarity to Neprylisin 11 from *Tribolium castaneum* (Table 2). Another transcript showed homology to Neprylisin 2 (DN12753_c1_g1_i4; LogFC = 10.8 compared to male thorax). Neprylisins are membrane-bound zinc metalloproteases that are responsible for the activation/inactivation of peptide hormones and neuropeptides^56^.

We also investigated the transcriptome of male fat body, an insect tissue possessing high metabolic and protein biosynthetic activity. The proteomics dataset of the *P. pyralis* male spermatophore contained several proteins that appear to be synthesized in male fat body (Table 2; Supplementary Table 3). One was a cysteine protease, Cathepsin L11 (DN10232_c0_g1_i1; LogFC = 2.2 compared to male thorax), a lysosomal endopeptidase that can be secreted and interact with structural proteins, such as collagen and fibronectin^57^.

### Metabolomic analysis of the firefly nuptial gift

To examine the small molecule composition of the *P. pyralis* spermatophore, we conducted an LC-HRAM-MS metabolomic analysis aimed at elucidating compounds specifically enriched in the spermatophore compared to extracts from the male body with the posterior abdomen excised. In an untargeted metabolomic analysis, we noted several mass features exclusively present or present at significantly higher abundance in the spermatophore extract. However, these mass features did not match any compounds in the KEGG Database, suggesting they may represent specialized metabolites yet to be identified (MetaboLights Supplementary Data).

**Figure 5:**
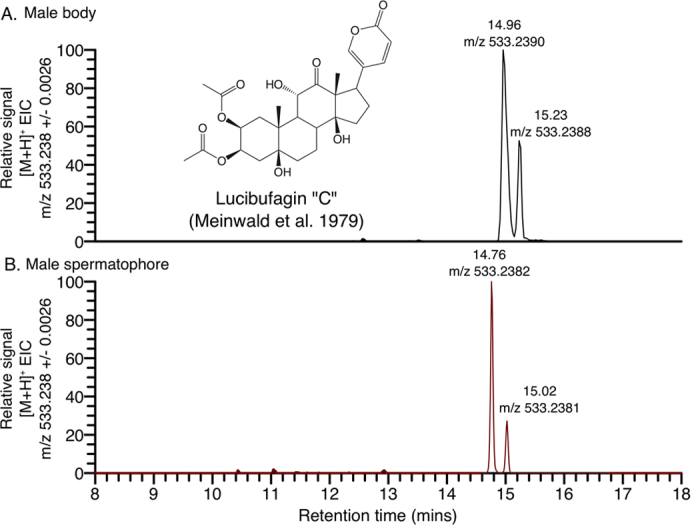
Positive ion mode extracted-ion-chromatograms (EIC) of the diacetylated lucibufagin [M + H]^+^ exact mass from an LC-HRAM-MS analysis of (**A**) *P. pyralis* male body (with posterior abdominal segments removed) and (**B**) *P. pyralis* male spermatophore. The difference in retention time is expected as these samples were run on different C18 liquid chromatography columns. The retention times of these features did match after retention time alignment (MetaboLights Supplementary Data).

Using a targeted metabolomic analysis, we determined that a known firefly defense compound, lucibufagin C, was present in both the spermatophore and male body. Lucibufagins have previously been shown to be a major class of anti-predator defense compounds in *Photinus* fireflies^58^,^59^. In the positive ion mode extracted ion chromatograms (EICs), both tissues showed a large peak characteristic of lucibufagin C, as well as a smaller second peak likely representing a different isomer of diacetylated lucibufagin (Fig. 5). This targeted analysis also identified *P. pyralis* pterin, a high-abundance compound of unknown function previously purified from *P. pyralis*^60^. The identity of lucibufagin C and *P. pyralis* pterin in the spermatophore was confirmed by comparison of retention time, exact mass, and MS/MS fragmentation spectra between male body and spermatophore. These compounds were among the most abundant mass features detected in the male body extract (Supplementary Fig. 3), and were identified without authentic chemical standards, as the feature retention time, exact mass, isotopic pattern, and fragmentation spectra were consistent with their respective structural identities.

### Gene expression in the female reproductive tract

To determine how specific tissues might process the spermatophore and interact with male reproductive proteins, we examined differential gene expression in the reproductive tract of *P. pyralis* females relative to thorax, although the single replicate available for female tissues meant that we could not test for statistical significance. However, using the more stringent criteria of a LogFC ≥ 3 and FDR < 0.01, we identified numerous highly expressed genes in different portions of the female reproductive tract (Table 3).

The female bursa copulatrix initially receives the male spermatophore, which is then moved into spermatophore-digesting gland where the spermatophore is degraded over several days. In the combined spermatophore-digesting gland and bursa tissues, we found 80 transcripts that were up-regulated compared to female thorax, of which 33 were annotated (Table 3). Four sequences showed homology to peptidases, including one sequence (DN8737_c0_g1_i1; LogFC = 4.9 compared to female thorax) with homology to angiotensin-converting enzyme, a zinc-metallopeptidase.

We also examined gene expression in the female spermatheca, where male sperm are stored prior to fertilization, and identified 80 up-regulated genes (39 annotated) in this female reproductive tissue (Table 3). As in the spermatophore-digesting gland and bursa, a sequence (DN8737_c0_g1_i1; LogFC = 6.2 compared to female thorax) with homology to angiotensin-converting enzyme was up-regulated in the female spermatheca, along with five other peptidases (Table 3). Another peptidase showed homology to Neprylisin 2.

## Discussion

Despite recent advances, we remain in the early stages of deciphering the molecular interactions that transpire between male ejaculates and the female reproductive tract during and after mating. Clearly, a necessary first step is to identify the players on both sides. To gain insight into postcopulatory sexual interactions, we sequenced the transcriptomes of both male and female reproductive glands in the firefly *P. pyralis* and performed proteomics and metabolomic analyses of the male spermatophore gift. Firefly spermatophores are produced by several distinct male reproductive glands, and are delivered to and processed within the female reproductive tract. Our *de novo* transcriptome of male reproductive glands demonstrated up-regulation of several proteases, which may play a role in postmating interactions, as well as transport proteins, which may serve to replenish seminal proteins transferred at mating. Combined with spermatophore bottom-up proteomics, we found 208 annotated proteins packaged into the *P. pyralis* male spermatophore and transferred to females, and identified the putative anatomical production sites for 68 of these male proteins. We also identified 217 spermatophore proteins that could not be annotated and may represent proteins that are rapidly evolving. Targeted metabolomic analysis also yielded the first evidence that *P. pyralis* males may incorporate lucibufagins, the primary antipredator defensive compounds in *Photinus* fireflies, into their nuptial gifts. We also examined gene expression in the female reproductive tract, and found up-regulation of several proteases. These results are discussed in greater detail below.

### Molecular Composition of Firefly Nuptial Gifts

Recent work reveals proteases to be a conserved protein class in the male seminal gifts of diverse taxa^18^, suggesting their proteolytic roles help to regulate postcopulatory interactions. This study demonstrates that the reproductive accessory glands of *P. pyralis* males synthesize serine proteases, metalloproteases, and cysteine proteases, many of which are packaged into the nuptial gift and delivered to females (Table 2).

We identified several metalloproteases that are produced by male accessory glands and transferred to females within the male spermatophore (Table 2; Supplementary Table 3). Metalloproteases transferred in seminal fluid of *D. melanogaster* have been linked to the induction of egg laying and are also important for spermatogenesis and fertilization^56^. Neprylisin 2, produced in *P. pyralis* male other accessory glands, has also been identified in male ejaculates of *Dermacentor variabilis* ticks^61^, and *Melanoplus sanguinipes* grasshoppers^62^. When Neprylisin*-like 1* was knocked down in male mice, their mates produced smaller litters^63^. Similarly, down-regulation of Neprylisin *2* in *D. melanogaster* males reduced post-mating fertility in females^64^. Angiotensin-converting enzyme, shown here to be transferred to females in the *P. pyralis* male spermatophore (Supplementary Table 4), has been shown to also occur in the seminal fluid of several insects, including *C. capitata* fruit flies^20^, *T. oceanicus*^53^ and *M. sanguinipes* grasshoppers^62^, and the flour beetle *T. castaneum*^65^. In *T. casteneum*, knockdown of angiotensin-converting enzyme in males led to the production of abnormal sperm and decreased egg production by their mates^65^. We also identified ADAMTS (DN15036_c0_g1_i8), another metalloprotease, which was up-regulated in the spiral glands of *P. pyralis* males (Fig. 3; Supplementary Table 2) and may be important for sperm fertilization ability^66^; however, this was not detected in our spermatophore proteomics. It is important to note that the detection threshold for a given protein with bottom-up proteomics varies greatly, hence the absence of a protein in our proteomic results does not rule out its presence in the spermatophore. Overall, several metalloproteases synthesized in *P. pyralis* male accessory glands may be important for increasing male fertilization success, perhaps through enhancing sperm storage and/or release.

Serine proteases represent common components of insect ejaculates, and have been shown to mediate post-mating physiological changes in females of several taxa. For example, trypsin-like serine proteases transferred in male nuptial gifts increase female oviposition in *A. socius* crickets^67^ and *D. melanogaster* fruit flies^18^. In *P. pyralis* males, the spiral glands produce two serine proteases, Snake and Trypsin 1, and we confirmed these are also transferred to females in spermatophores (Table 2). Snake acts as an important mediator of the Toll pathway immune response in mosquitoes and fruit flies and is part of a serine protease cascade controlling synthesis of drosomycin, an antifungal agent^68^,^69^,^70^. Thus, inclusion of these enzymes in *P. pyralis* nuptial gifts may potentially increase female immune response and reduce the likelihood of infection by microbial pathogens introduced during mating.

We also identified cysteine protease, Cathepsin L 11, a papain-like enzyme that appears to be produced by male fat body and transferred to the female (Table 2). Cathepsin L is a secreted lysosomal endopeptidase, which degrades structural proteins such as collagen and fibronectin^71^. Cathepsins are responsible for digestive proteolysis in the gut of cowpea weevils *Callosobruchus maculatus*^72^, and may play a similar role in degrading the male spermatophore inside the spermatophore-digesting gland. Another possible function is suggested by high concentrations of Cathepsin L found in pre-ovulatory follicles of mice where it may initiate follicular rupture and ovulation^73^. Radiolabeling studies in other *Photinus* fireflies demonstrated that some spermatophore-derived proteins are incorporated into female oocytes^45^, so inclusion of Cathepsin L in the *P. pyralis* nuptial gift may act to stimulate follicle degradation and ovulation.

Certain *Photinus* fireflies are known to derive protection against their predators through biosynthesis of specialized toxic steroidal pyrones known as lucibufagins^58^,^59^. Notably, our metabolomic analysis provides preliminary evidence that *P. pyralis* males transfer detectable quantities of lucibufagins to females in their nuptial gift (Fig. 5; Supplementary Table S3). We hypothesize that males may package lucibufagins into their nuptial gift, where these defense compounds could augment the female’s own defenses to help protect the female or her eggs against predators or microbial attack. Previous studies have found that other insect males also transfer defensive chemicals to females within their spermatophores or seminal fluid^8^. In many cases, such defensive compounds are derived from host plants, including pyrrolizidine alkaloids in *Utetheisa ornatrix* moths^74^, cyanogenic glycosides in several *Heliconius* butterflies^75^, and vicilin-derived peptides in *Callosobruchus maculatus* cowpea beetles^76^. In blister beetles (family Meloidae), however, males actively synthesize a toxic terpene, cantharidin, which they store in their major accessory glands and transfer in their nuptial gifts^77^. In fireflies, further experiments are needed to definitively elucidate the source of male lucibufagins, to quantify amounts contained within male nuptial gifts, and to determine the extent to which male-derived lucibufagins may be used to defend the female or her eggs.

### Gene Expression in Female Reproductive Tracts

Postcopulatory sexual interactions are evolutionarily important, yet have proven challenging to study because they typically take place within the female reproductive tract. Moreover, to date, gene expression in those female reproductive tissues that receive and process male ejaculates has been examined for only a few taxa, including *Drosophila* spp.^27^, the honeybee *Apis mellifera*^30^, the corn borer moth *Ostrinia nubilalis*^25^, and *Pieris rapae* butterflies^26^. In *Photinus* fireflies, after the male spermatophore is deposited in the female’s bursa copulatrix, it enters the spermatophore-digesting gland where it subsequently disintegrates over the next several days^40^. Male sperm are stored and remain viable within the female’s spermatheca for up to two weeks before fertilization^78^. In this study of *P. pyralis* fireflies, we demonstrated that the sperm- or spermatophore-receiving portions of the female reproductive tract express genes encoding proteases, protease inhibitors, and other proteins involved in immune response and in maintaining sperm viability.

Female peptidases and peptidase regulators are likely to be important mediators of postcopulatory sexual interactions, and these have also been identified from female reproductive tracts of other insects^25^,^26^,^27^. In *D. melanogaster*, female peptidases and peptidase inhibitors interact with male SFPs and are required to process into their active forms at least three male proteins that induce egg-laying and reduce female receptivity to remating^79^. Within the *Drosophila repleta* group, females peptidases and peptidase inhibitors expressed in more promiscuous species show higher dN/dS ratios compared to monogamous species, indicating strong positive selection on these female reproductive proteins^80^.

In this study, we identified several proteases that are expressed in the reproductive tract of *P. pyralis* females. In the spermatheca, we identified six peptidases, including *N*eprilysin 2 (DN42525_c0_g1_i1), that were up-regulated compared to the female thorax. In both female tissues, we found a sequence encoding angiotensin-converting enzyme (DN8737_c0_g1_i1) that was up-regulated compared to the female thorax. As in male insects, female neprilysins have been shown to be critical in maintaining *D. melanogaster* fertility^64^. When *N*eprilysin *2* is knocked down in *D. melanogaster* females, fewer eggs are laid and they show decreased viability^64^. In *P. pyralis* females, *N*eprilysin 2 may also play a role in regulating ovulation and maintaining egg viability. Angiotensin-converting enzyme was also found in both *P. pyralis* female tissues, and has previously been shown to be expressed in the bursa copulatrix and spermatheca of female *Lacanobia oleracea* moths^81^. The function of this peptidase in the female reproductive tract has yet to be determined.

In summary, this study offers new insights into the molecular composition of the firefly spermatophore, and deepens our understanding of how such nuptial gifts can mediate postcopulatory interactions between males and females. One future challenge will be to perform functional studies in fireflies and other non-model organisms to determine how these reproductive proteins influence reproductive fitness of both sexes. Future studies examining intraspecific differences in nuptial gift composition will also shed light on the evolutionary forces that drive the origin and maintenance of nuptial gifts across taxa.

## Materials and Methods

### Specimen and tissue collection

*Photinus pyralis* fireflies used in this study were collected at Mercer Meadows Pole Farm, Lawrenceville, NJ (40°18′23.4″N, 74°44′53.9″W) on 27 June and 11–12 July 2015, and identified based on male genitalia^82^ and flash patterns. Both sexes were kept individually in plastic containers with sliced apple and damp paper towel. Mating status of field-collected individuals was unknown. Fireflies were kept in the lab for less than one week prior to experimentation.

We compared gene expression in male and female reproductive tissues as shown in Supplementary Fig. 2. Tissues were collected from fireflies anesthetized at −20 °C for 20 min then dissected under 40–70x magnification in RNAlater. From 12 *P. pyralis* males, the following tissues were dissected and pooled into 3 biological replicates (4 males each): spiral accessory glands, other accessory glands, thorax, and fat body. Insect fat body is a metabolically active tissue responsible for protein synthesis; widely distributed throughout the abdomen, fat body is abundant surrounding the male accessory glands. As fewer females were available, three females were pooled to produce a single biological replicate of the following tissues: spermatheca, spermatophore digesting gland and bursa, and thorax. All tissues were stored in RNAlater at −80 °C until RNA extraction.

After mating, *Photinus* males immediately begin assembling a new spermatophore^40^, thus we predicted that accessory glands of recently mated males would show higher transcription levels of functionally related genes. Males were mated with females in the lab, and then spiral accessory glands and other accessory glands were dissected 2 h after mating pairs had separated. Tissues harvested from four recently mated males were pooled into one biological replicate and stored in RNAlater at −80 °C until RNA extraction.

### RNA extraction and sample preparation

Prior to RNA extraction, each pooled biological replicate was frozen in liquid nitrogen and homogenized in QIAzol lysis reagent (Qiagen, Valenica CA USA) using a mortar and pestle. RNA was extracted using RNeasy Lipid Tissue Kit (Qiagen), and Illumina sequencing libraries were prepared from total RNA enriched to mRNA with a polyA pulldown using the TruSeq RNA Library Prep Kit v2 (Illumina, San Diego, CA). A total of 18 libraries were sequenced at the Whitehead Institute Genome Technology Core (Cambridge, MA) on two lanes of an Illumina HiSeq 2500 using rapid mode (PE 100 bp). Raw sequencing data has been uploaded to NCBI SRA (SRP078386).

### Transcriptome assembly and differential expression analysis

Resulting RNA-Seq reads in FASTQ format were checked with the FastQC software package (http://www.bioinformatics.babraham.ac.uk/projects/fastqc/), and Illumina TruSeq3 adaptor contamination and low quality reads were removed by the Trimmomatic software package (http://www.usadellab.org/cms/?page=trimmomatic)^83^, with the following parameters “ILLUMINACLIP:TruSeq3-PE.fa:2:30:10 SLIDINGWINDOW:4:5 LEADING:5 TRAILING:5 MINLEN:25”. 185,402,330 paired reads pooled from all libraries remained post quality filtering. A *de novo* transcriptome was assembled from the pooled quality-filtered paired reads with Trinity 2.2.0^84^ using default parameters with the exception of “–min_glue 5 –min_kmer_cov 3”, on a single high-memory server (Whitehead Institute). Candidate ORFs were translated *in silico* from the *de novo* transcriptome using Transdecoder 2.0.1^85^, with the minimum protein length set to 20 amino acids. The *de novo* transcriptome and predicted ORF annotations have been uploaded to NCBI TSA (GEZM00000000). We note that the sequence names of the uploaded sequences have an internal sequencing run and assembly version indicating prefix, “151_Ppyr_v3*_*TRINITY_”, but otherwise represent identical transcripts to those analyzed in the manuscript.

Expression analysis was performed using Trinity by the included “align_and_estimate_abundance.pl” script, with default parameters. This script utilizes Bowtie^86^,^87^ and RSEM^88^ to map reads to assembled transcripts and perform transcript quantification with expectation maximization respectively. We identified male and female genes that were significantly differentially expressed between specific tissues using the Bioconductor package edgeR (comparisons of interest shown in Supplementary Fig. 2 89,90,91,92. Male genes were considered significantly differentially expressed if they had a log_2_ fold change ≥ 2 (logFC) and a false discovery rate (FDR) ≤ 0.01. We focused our subsequent analysis on male genes that showed significant up-regulation in either spiral accessory glands or other accessory glands relative to male thorax. Female comparisons lacked replicates, so significant differential expression could not be assessed. Because of this lack of replication for female tissues, we are cautious in our conclusions drawn from this data. We also compared genes that were up-regulated compared to thorax in male spiral accessory glands (LogFC ≥ 10) to those up-regulated in male other accessory glands compared to male thorax (LogFC ≥ 10) directly. This list of genes was then analyzed for differential expression between the spiral accessory glands and the other accessory glands to determine how the function of each tissue differs.

After differential expression analysis, all significantly differentially expressed genes were annotated using Blast2GO and InterProScan^93^,^94^,^95^. To identify putative homologs, a Blast search was conducted between each sequence and the entire NCBI non-redundant protein database^96^. All sequences with significant Blast hits (e-value ≤10^−10^) were then mapped and annotation scores were computed for all possible gene ontology terms. We used InterProScan to obtain further protein domain/motif information, enabling us to identify protein domains that indicated secretion^97^. It is important to note, these annotation methods rely on previously characterized proteins, making it difficult to annotate more rapidly evolving sequences. Here, we only discuss sequences that that were successfully annotated using Blast2GO. All differentially expressed genes that were successfully annotated had similarity search e-values ≤ 6 × 10^−10^.

### Principal component analysis

We summarized multivariate variation in gene expression among various male and female tissues using principal component analysis. To normalize read counts, the trimmed mean of M-values normalization method was conducted for each transcript using edgeR^89^,^90^,^91^,^98^. Next a biological coefficient of variation analysis was conducted in edgeR.

### Spermatophore proteomics

One hour after the initiation of stage II copulation^99^, a mating pair of *Photinus pyralis* fireflies was separated, and the spermatophore was carefully dissected out from the female’s reproductive tract. Upon removal from storage, the spermatophore was transferred into 50 μL of 2× Laemmli Sample Buffer (Bio-Rad) with 2% β-mercaptoethanol, and heated to 95 °C for 5 min. Sample (25 μL) was loaded onto a 12% percent discontinuous Laemelli SDS-PAGE gel. BLUEstain™ Protein ladder (Gold Biotechnology) was loaded in a neighboring well for inferring protein size. Eight sections containing proteins ranging from >180 kDa to ~6 kDa were cut from the gel, and provided to the Whitehead Institute Proteomics Core Facility (Cambridge, MA). Thereafter the samples were digested with trypsin, and run individually on a Dionex Ultimate 3000 RSLCnano nanoflow LC coupled to a ThermoFisher Scientific Orbitrap Elite mass spectrometer. *In silico* translated ORFs from the Trinity *de novo* transcriptome concatenated with common contaminants in proteomics were used as the search database to identify tryptic peptides from the samples. Mascot (Matrix Science, London, UK; version 2.5.1) was used as the proteomic search engine. Verification of peptide and protein identification and general analysis was performed in Scaffold (Supplementary methods 1; Scaffold version 4.4.8, Proteome Software Inc., Portland, OR). Raw proteomic data and peptide identifications have been uploaded to the EBI PRIDE database (https://www.ebi.ac.uk/pride/archive/) with the following accession number (PXD004005). Potential signal peptides were predicted from the in-silico predicted ORFs using SignalP-4.1^100^.

### Spermatophore metabolomics

To examine the small molecule composition of the male spermatophore, we conducted an untargeted liquid-chromatography high-resolution accurate-mass mass-spectrometry (LC-HRAM-MS) metabolomic analysis aimed at elucidating compounds specifically enriched in the spermatophore. Again, a pair *P. pyralis* fireflies was separated shortly after mating, and the spermatophore carefully dissected out of the female’s reproductive tract. Briefly, we compared mass features detected in 1:1 water:methanol extracts of the spermatophore and the body of an adult *P. pyralis* male whose posterior abdominal segments (including the lantern and reproductive tissues) had been removed. We conducted targeted analyses to look for lucibufagin, pterin, and several insect hormones, as well as an untargeted metabolomic analysis to identify any compounds enriched in male spermatophores. Data processing and analysis was performed with MZmine2^101^ (see Supplementary Methods for details). Raw and mzTab format feature called metabolomic data from the *P. pyralis* spermatophore and body have been uploaded to the EBI MetaboLights database (http://www.ebi.ac.uk/metabolights/) with the following accession number (MTBLS362).

## Additional Information

**How to cite this article**: Al-Wathiqui, N. *et al*. Molecular characterization of firefly nuptial gifts: a multi-omics approach sheds light on postcopulatory sexual selection. *Sci. Rep.* **6**, 38556;, doi: 10.1038/srep38556 (2016).

**Publisher's note:** Springer Nature remains neutral with regard to jurisdictional claims in published maps and institutional affiliations.

## Supplementary Material

Supplementary Information

## Figures and Tables

**Figure 1:**
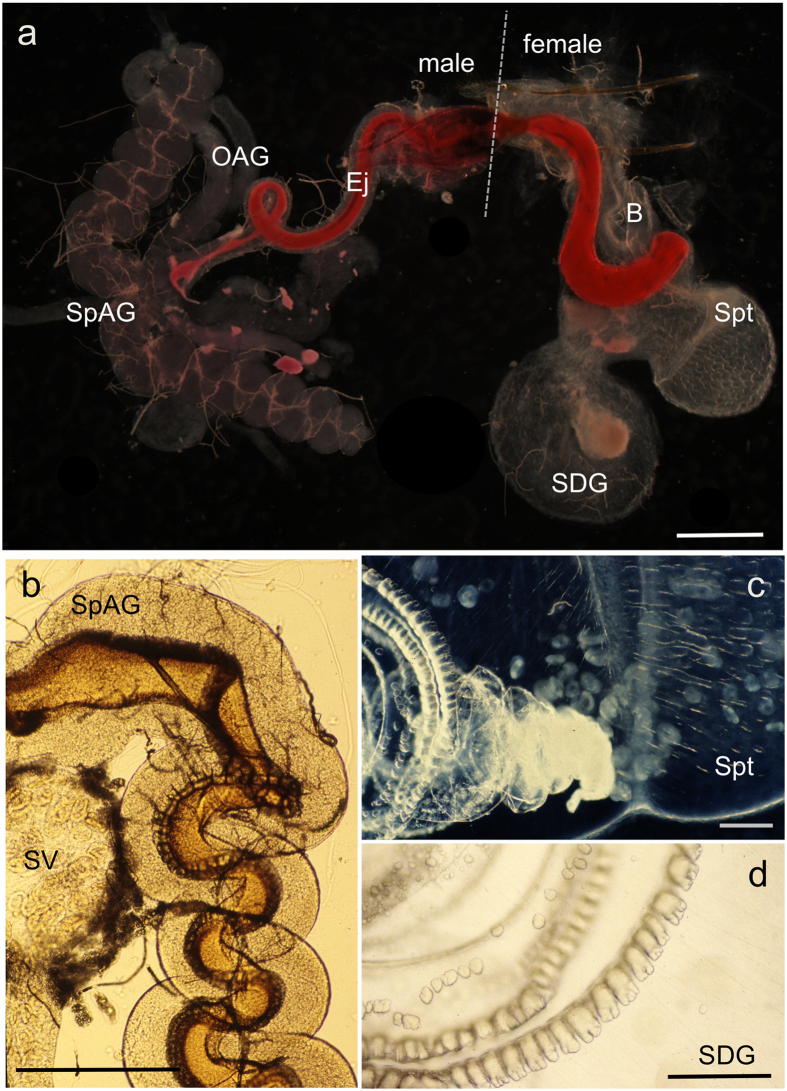
Nuptial gift formation, transfer and fate in *Photinus* fireflies. (**a**) During mating the male spermatophore (stained here with rhodamine B) moves through the ejaculatory duct (Ej) into the female’s bursa copulatrix (B). Several male glands contribute to the spermatophore, including the paired spiral glands (SpAG), and other accessory glands (OAG; long accessory gland not shown). (**b**) Spiral accessory glands (SpAG) manufacture the major portion of the spermatophore, which is visible as a dark structure edged with serrated scales; seminal vesicle (SV) stores sperm rings that get packaged into the spermatophore before transfer. (**c**) After transfer, sperm released from the tip of the spermatophore enter the female spermatheca (Spt), the sperm storage organ; the clear spermatophore sheath is visible (originally published in ref. 34). (**d**) The rest of the spermatophore moves into the spermatophore-digesting gland (SDG) where it disintegrates over the next 2–3 d). Scale bars are 500 µm (**a**,**b**) and 50 µm (**c**,**d**).

**Table 1:** GO categories describing molecular function of genes expressed in reproductive accessory glands of *P. pyralis* males.

Proposed functional class	Sequences unique to OAGs* (%, n)	Sequences unique to SpAGs^†^ (%, n)
Transmembrane transport	3%, 6	3%, 2
Peptidases and Peptidase Regulation	8%, 14	0
Signal transduction	0.5%, 1	0
Developmental proteins	1%, 2	4%, 3
Unknown conserved proteins	0.5%, 1	0
General cellular processes	3%, 6	8%, 6
Novel	83%, 151	85%, 64

*Other accessory glands (OAGs).

^†^Spiral accessory glands (SpAGs).

**Table 2:** Transcripts encoding spermatophore proteins and their proposed tissue of production in *P. pyralis* males.

Tissue and protein functional class	Sequence ID-description	e-value	% similarity	MW (kDa)	Gel Section	Predicted Signal Peptide?
**Spiral Accessory Gland (SpAG)**						
Peptidases and peptidase regulators						
	DN8730_c0_g1_i1- trypsin 1 like	2.4 × 10^−58^	61.00%	28	5	+
	DN10938_c0_g1_i1- serine protease snake	1.1 × 10^−117^	70.80%	33	5	+
Unknown conserved proteins						
	DN13942_c0_g1_i1- uncharacterized protein LOC656585	0	92.40%	76	3	+
General cellular processes						
	DN11025_c0_g1_i1- Histone 2B	4.0 × 10^−58^	99.20%	14	7	
	DN13259_c0_g1_i1-60 kDa heat shock protein	0	95.30%	61	3	
	DN8371_c0_g1_i1- elongation factor 1-alpha	0	95.30%	50	4	
	DN12873_c0_g1_i2-40S ribosomal S9	1.3 × 10^−123^	99.50%	23	5	
	DN13943_c0_g1_i1- eukaryotic translation initiation factor 5 A	5.0 × 10^−99^	98.10%	18	7	
	DN1419_c0_g1_i1-40S ribosomal S13	1.2 × 10^−95^	97.20%	17	7	
**Other Accessory Glands (OAG)**						
Peptidases and peptidase regulators						
	DN14826_c0_g1_i1- neprilysin-11	0	83.80%	89	2	
	DN17149_c1_g1_i1- aminopeptidase N	0	65.30%	105	2	+
	DN12753_c1_g1_i4- neprilysin 2	2.0 × 10^−104^	72.70%	31	5	
Structural component of cell						
	DN19550_c0_g1_i1- glycine rich cell wall structural	7.3 × 10^−19^	65.70%	31	5	+
	muscle specific protein 20	3.3 × 10^−111^	92.30%	20	6	
General cellular processes						
	DN11298_c0_g1_i1- annexin B10	7.7 × 10^−144^	80.80%	36	4	
	DN1632_c0_g1_i1- V-type proton ATPase subunit G	4.2 × 10^−33^	94.80%	14	7	
	DN15081_c0_g1_i1- heat shock 70 kDa cognate 3	0	97.60%	72	3	+
	DN16493_c0_g1_i1- glutamate dehydrogenase	0	90.10%	61	3	
	DN7056_c1_g1_i1- arylsulfatase B	0	78.90%	58	4	+
	DN17778_c0_g2_i6- insulin-like growth factor-binding complex	4.6 × 10^−25^	52.60%	29	5	
	DN13283_c0_g1_i1- V-type proton ATPase subunit A	0	97.20%	68	3	
	DN1410_c0_g1_i1- subunit B	0	99.00%	55	4	
	DN54_c0_g1_i1- disulfide-isomerase A3	0	85.80%	55	4	+
**Fat body**						
Peptidases and peptidase regulators						
	DN10232_c0_g1_i1- cathepsin L11	0	81.70%	63	3	+
General cellular processes						
	DN15562_c0_g1_i1- beta-galactosidase	0	79.30%	75	3	

**Table 3:** Transcripts encoding female reproductive genes and their annotation description in *P. pyralis* fireflies.

Tissue and category	Sequence ID - description	e-value	% similarity	Predicted signal peptide?
**Spermatophore-digesting gland (SDG) and Bursa (B)**				
Peptidases and peptidase regulators				
	DN17891_c2_g1_2- protease RNA-dependent RNA partial	7.2 × 10^−36^	73.90%	
	DN10767_c0_g1_i1- protease/RNA-dependent RNA polymerase	7.6 × 10^−41^	82.60%	
	DN15516_c0_g1_i5- dipeptidyl peptidase 9	0	93.10%	
	DN8737_c0_g1_i1- angiotensin-converting enzyme	0	89.90%	
Transport				
	DN12916_c0_g1_i1- solute carrier family 12 member 6 isoform X7	0	85.00%	
	DN15627_c0_g1_i5- plasma membrane calcium-transporting ATPase 2	0	94.10%	
	DN5842_c0_g1_i1- innexin inx1	0	88.90%	
Development				
	DN17388_c0_g1_i4- suppressor of hairless	0	96.10%	
	DN15473_c0_g1_i4- homeobox abdominal-B-like isoform X3	4.6 × 10^−77^	70.40%	
Unknown conserved proteins				
	DN9101_c0_g1_i1- uncharacterized protein LOC664274	6.1 × 10^−58^	83.00%	
General cellular processes				
	DN13365_c0_g1_i3- zinc finger 271-like	0	62.10%	
	DN16484_c0_g1_i2- DNA helicase MCM9	0	82.80%	
	DN16354_c1_g1_i2- splicing factor 1	1.1 × 10^−133^	98.10%	
	DN12336_c0_g1_i1- E3 ubiquitin- ligase TRIM37-like	0	72.60%	
	DN13021_c0_g1_i1- Mitochondrial dicarboxylate carrier	2.2 × 10^−145^	85.30%	
	DN17027_c2_g1_i5- eukaryotic translation initiation factor 2-alpha kinase 4	1.0 × 10^−76^	49.80%	
	DN12815_c0_g2_i3- dihydropyrimidinase isoform X1	8.9 × 10^−174^	88.50%	
	DN10803_c0_g1_i2- cyclin-L1 isoform X2	2.6 × 10^−109^	80.70%	
	DN12251_c0_g1_i1- carnitine O-palmitoyltransferase mitochondrial	0	71.50%	
	DN13981_c0_g1_i2- ESF1 homolog	0	75.00%	
	DN13659_c0_g1_i5- RNA binding protein	2.9 × 10^−164^	98.40%	
	DN17535_c1_g4_i3- Sphingomyelin phosphodiesterase	1.5 × 10^−75^	73.00%	
	DN17637_c0_g2_i5- acyl-synthetase family member 4 isoform X3	2.2 × 10^−132^	59.60%	
	DN15346_c0_g1_i1- alkaline phosphatase 4-like	8.4 × 10^−178^	67.60%	+
	DN10122_c0_g2_i1- alpha-L-fucosidase	0	79.30%	
	DN11997_c0_g1_i1- polyprotein	7.7 × 10^−68^	49.80%	
	DN17891_c2_g1_i1- polyprotein	2.1 × 10^−112^	66.20%	
	DN12512_c0_g1_l- polyprotein	4.0 × 10^−57^	66.40%	
	DN15814_c0_g1_i1- polyprotein	1.5 × 10^−66^	76.70%	
	DN13377_c0_g1_i1- polyprotein	3.8 × 10^−31^	31.70%	
	DN8502_c0_g1_i1- polyprotein	3.3 × 10^−24^	56.60%	
	DN454_c0_g1_i1- polyprotein	2.7 × 10^−25^	56.80%	
	DN17327_c1_g1_i3- blastopia polyprotein	0	63.70%	
**Spermatheca** (**Spt**)				
Peptidases and peptidase regulators				
	DN16681_c1_g1_i1- probable aminopeptidase NPEPL1	0	87.70%	
	DN17891_c2_g1_i2- protease RNA-dependent RNA partial	7.2 × 10^−36^	73.90%	
	DN8737_c0_g1_i1- angiotensin-converting enzyme	0	89.90%	
	DN42525_c0_g1_i1- neprilysin 2	0	89.50%	
	DN10767_c0_g1_i1- protease RNA-dependent RNA partial	7.6 × 10^−41^	82.60%	
	DN15516_c0_g1_i5- dipeptidyl peptidase 9	0	93.10%	
Chitin metabolism				
	DN5290_c0_g1_i1- chitin deacetylase 1 precursor	0	96.50%	+
Structural/components of membrane				
	DN16210_c0_g1_i11- inward rectifier potassium channel 2-like isoform X1	0	82.10%	
	DN15532_c0_g1_i2- Aquaporin	2.5 × 10^−98^	80.60%	
	DN2886_c0_g1_i1- lethal(3)malignant blood neoplasm 1	9.4 × 10^−52^	82.90%	
Transport				
	DN12916_c0_g1_i1- solute carrier family 12 member 6 isoform X7	0	85.00%	
	DN15627_c0_g1_i3- plasma membrane calcium-transporting ATPase 2 isoform X1	0	95.80%	
	DN15627_c0_g1_i5- lasma membrane calcium-transporting ATPase 2 isoform X1	0	94.10%	
Development				
	DN17388_c0_g1_i4- suppressor of hairless	0	96.10%	
	DN15473_c0_g1_i8- homeobox abdominal-B isoform X1	3.8 × 10^−80^	76.40%	
	DN15473_c0_g1_i4- homeobox abdominal-B-like isoform X3	4.6 × 10^−77^	70.40%	
	DN17215_c0_g5_i5- homeobox extradenticle isoform X3	6.2 × 10^−178^	84.00%	
Unknown conserved proteins				
	DN9101_c0_g1_i1- uncharacterized protein LOC664274	6.1 × 10^−58^	83.00%	
General cellular processes				
	DN17737_c0_g1_i2- zinc finger 106	2.2 × 10^−74^	61.20%	
	DN15941_c0_g1_i5- tetratricopeptide repeat 14 homolog isoform X2	7.4 × 10^−110^	86.20%	
	DN16354_c1_g1_i2- splicing factor 1	1.0 × 10^−133^	98.10%	
	DN13283_c0_g1_i1- V-type proton ATPase catalytic subunit A	0	97.20%	
	DN11767_c0_g2_i2- V-type proton ATPase subunit H isoform X1	0	92.30%	
	DN17027_c2_g1_i5- eukaryotic translation initiation factor 2-alpha kinase 4	8.6 × 10^−79^	41.70%	
	DN15391_c2_g1_i2- four and a half LIM domains 2 isoform X7	8.2 × 10^−134^	92.80%	
	DN10803_c0_g1_i2- cyclin-L1 isoform X2	2.6 × 10^−109^	80.70%	
	DN7056_c1_g1_i1- arylsulfatase B	0	78.90%	+
	DN17637_c0_g2_i5- acyl-synthetase family member 4 isoform X3	2.2 × 10^−132^	59.60%	
	DN15346_c0_g1_i1- alkaline phosphatase 4-like	8.5 × 10^−178^	67.60%	+
	DN17839_c12_g9_i4- adenosylhomocysteinase 2 isoform X2	2.3 × 10^−146^	98.20%	
	DN12336_c0_g1_i1- E3 ubiquitin-ligase TRIM37-like	0	72.60%	
	DN13021_c0_g1_i1- mitochondrial dicarboxylate carrier	2.2 × 10^−145^	85.30%	
	DN17719_c0_g1_i1- ATP-binding cassette sub-family G member 8	0	84.50%	
	DN12765_c0_g1_i4- PAB-dependent poly(A)-specific ribonuclease subunit PAN3	0	88.40%	
	DN11997_c0_g1_i1- polyprotein	7.7 × 10^−68^	76.70%	
	DN17891_c2_g1_i1- polyprotein	2.1 × 10^−112^	73.60%	
	DN12512_c0_g1_i1- polyprotein	3.9 × 10^−57^	66.20%	
	DN15814_c0_g1_i1- polyprotein	1.5 × 10^−66^	49.80%	
	DN17327_c1_g1_i3- blastopia polyprotein	0	63.10%	
