# Supplementary material for: Molecular characterization of firefly nuptial gifts: a multi-omics approach sheds light on postcopulatory sexual selection
Source: Sci Rep. 2016 Dec 22;6:38556. doi: 10.1038/srep38556 (PMC5177949; doi:10.1038/srep38556)
Supplement: Supplementary Information [file srep38556-s1.docx]

Supplemental Material

Molecular characterization of firefly nuptial gifts: a multi-omics approach sheds light on postcopulatory sexual selection

**Nooria Al-Wathiqui^1*^, Timothy R. Fallon ^2,3*^, Adam South ^4^, Jing-Ke Weng^2,3✝^,** **Sara M. Lewis^1✝^**

^1^ Department of Biology, Tufts University, Medford MA 02155, USA

^2^ Whitehead Institute for Biomedical Research, 9 Cambridge Center, Cambridge MA 02142, USA

^3^ Department of Biology, Massachusetts Institute of Technology, Cambridge MA 02139 USA

^4^ Department of Immunology and Infectious Disease, Harvard T.H. Chan School of Public Health, Boston MA 02115, USA

^*^ co-first authors

corresponding authors:

Jing-Ke Weng, wengj@wi.mit.edu

Sara M. Lewis, [sara.lewis@tufts.edu](mailto:sara.lewis@tufts.edu)

**Supplemental Methods: Bottom-up Proteomics**

DATABASE SEARCHING-- Tandem mass spectra were extracted, charge state deconvoluted and deisotoped by the Mascot ExtractMSn utility. All MS/MS samples were analyzed using Mascot (Matrix Science, London, UK; version 2.5.1). Mascot was set up to search translated ORFs from the de-novo transcriptome concatenated with common contaminants (45515 entries) assuming the digestion enzyme stricttrypsin. Mascot was searched with a fragment ion mass tolerance of 0.60 Da and a parent ion tolerance of 20 PPM. Carbamidomethyl of cysteine was specified in Mascot as a fixed modification and oxidation of methionine as a variable modification.

CRITERIA FOR PROTEIN IDENTIFICATION-- Scaffold (version Scaffold_4.4.8, Proteome Software Inc., Portland, OR) was used to validate MS/MS based peptide and protein identifications. Peptide identifications were accepted if they could be established at greater than 95.0% probability by the Peptide Prophet algorithm (Keller, A et al Anal. Chem. 2002;74(20):5383-92) with Scaffold delta-mass correction. Protein identifications were accepted if they could be established at greater than 99.9% probability and contained at least 3 identified peptides. Protein probabilities were assigned by the Protein Prophet algorithm (Nesvizhskii, Al et al Anal. Chem. 2003;75(17):4646-58). Proteins that contained similar peptides and could not be differentiated based on MS/MS analysis alone were grouped to satisfy the principles of parsimony. Proteins sharing significant peptide evidence were grouped into clusters.

**Supplementary Methods: LC-HRAM-MS Metabolomics**

*Tissue processing plus extraction:*

A single spermatophore dissected from a male Photinus pyralis was placed in 100 µL of 50% methanol. A single adult male Photinus pyralis was flash frozen in liquid nitrogen, and the posterior 2 abdominal segments (containing lantern & genetalia) were removed with a razor blade at 4 ˚C. The remaining anterior portion of the firefly, hereafter called the “body”, was placed in 150 µL 50% MeOH. Both tissues were macerated in the solvent, and intermittently sonicated in a water bath sonicator for 30 minutes, not letting the temperature rise above 40 ˚C. Post sonication, the extract was centrifuged in a benchtop centrifuge at 14,000 g @ 4˚C for 10 min to pellet tissue debris and other particulates. The clarified extract was filtered through a 0.2 µm PFTE filter (Filter Vial, P/No. 15530-100, Thomson Instrument Company).

*Liquid chromatography:*

20 µL of the filtered extracts were separated on a UltiMate 3000 (Dionex) HPLC by reversed-phase chromatography on a 150 mm C18 Column (Kinetex 2.6 µm silica core shell C18 100Å pore, P/No. 00F-4462-Y0, Phenomenex), utilizing a gradient of Solvent A (0.1% formic acid in H2O) and Solvent B (0.1% formic acid in acetonitrile); 5% B for 2 min, 5-80% B over 40 min, 95% B for 4 min, and 5% B for 5 min; flow rate 0.8 mL/min. The flow from this chromatography was coupled to a Q-Exactive (Thermo-Scientific) mass spectrometer.

*Mass spectrometer settings:*

The Q-Exactive mass spectrometer was configured to perform 1 MS1 scan from m/z 120-1250 followed by 1-3 data-dependent MS2 scans using HCD fragmentation with a stepped collision energy of 10, 15, 25 normalized collision energy (NCE). Data was collected as profile data. The instrument was always used within 7 days of the last mass accuracy calibration. The ion source parameters were as follows: spray voltage (+) at 3000 V, spray voltage (-) at 2000 V, capillary temperature at 275 ˚C, sheath gas at 40 arb units, aux gas at 15 arb units, spare gas at 1 arb unit, max spray current at 100 (µA), probe heater temp at 350 ˚C, ion source: HESI-II.

*Data processing:*

The raw profile data in Thermo format was converted to mzML format using ProteoWizard MSConvert (Chambers et al. 2012) with 64-bit binary encoding precision, index writing, gzip compression of the whole file, zlib compression of peaklist data , and numpress linear compression of peaklist data. Data analysis was performed with MZmine2 2.19 (Pluskal et al. 2010) and Xcalibur 2.2 SP1.48 (Thermo Scientific).

*Metabolite feature processing:*

1. Thermo .raw data was converted to .mzML by ProteoWizard MSConvert with the parameters specified above.

2. Raw data was imported from .mzML format files.

3. MS^1^ and MS^2^ profile mass spectra were detected/centroided with the “Mass Detector” module. The following parameters were used:

- Mass Detector: Exact mass

- Noise level: 1E4

4. Continuous MS^1^ ions were assembled into mass traces with the “Chromatogram builder” module. The following parameters were used:

- Minimum time span (min): 0.1 the

- Minimum height: 1.0E4, and the

- m/z tolerance: 0.002 or 5ppm (whichever is greater)

5. Chromatograms were then deconvolved into individual peaks, using the “Chromatogram deconvolution” module, with the algorithm set to “Local minimum search” and following parameters:

- Chromatographic threshold: 1.0%

- Search minimum in RT range (min): 0.1

- Minimum relative height: 0.5%

- Minimum absolute height: 1.0E3

- Min ratio peak top/edge: 4

- Peak duration range (min): 0.0 – 5.0

6. The MS^1^ mass spectra were then deisotoped with the “Isotopic peak grouper” module. The following parameters were used:

- m/z tolerance: 0.01025 Da or 20ppm (whichever is greater)

- Retention time tolerance: 0.2 (absolute (min))

- Monotonic shape: unchecked

- Maximum charge: 2

- Representative isotope: Most intense

7. Mass features were aligned with a correction for retention time deviation between the *Photinus pyralis* body and spermatophore extract, using the “RANSAC aligner” module with the following parameters:

- m/z tolerance: 8.0E-4 or 4ppm (whichever is greater)

- RT tolerance: 2.0%

- RT tolerance after correction: 0.25 ( absolute (min) )

- RANSAC iterations: 1000000

- Minimum number of points: 30.0%

- Threshold value: 0.08 ( absolute (min) )

- Linear model: unchecked

- Require same charge state: unchecked

8. Putative in-source fragments were identified by the “Fragment search” module with the following parameters:

- Retention time tolerance: 0.3 (absolute (min) )

- m/z tolerance of MS2 data: 0.001 or 5ppm (whichever is greater)

- Max fragment peak height: 50%

- Min MS2 peak height: 1.0E5

9. Putative adduct ions were identified by the “Adduct search” module

with the following parameters:

- Retention time tolerance: 0.3 (absolute (min) )
- m/z tolerance of MS2 data: 0.0005 or 2ppm
- Max relative adduct peak height: 20%
- The following default MZmine2 adducts were selected:
  - [M+Na-H]
  - [M+K-H]
  - [M+NH3]

10. Putative complexed ions were detected by the “Complex search” module with the following parameters:

- Retention time tolerance: 0.3 (absolute (min) )

- m/z tolerance of MS2 data: 0.001 or 5ppm (whichever is greater)

- Max complex peak height: 80.0%

11. The resulting peaklist was exported as “spermatophore_vs_body_final_unfiltered.mzTab” (Supplementary MetaboLights Data - MTBLS362)

12. The peaklist was filtered to remove putative complexes, adducts, and fragments, using the “peaklist rows filter” module, with the custom parameters:

- Text in identity: “Adduct” or “Complex” or “Fragment”

- Keep or remove rows: “Remove rows that match all criteria”

This step used a custom fork of MZmine2 2.1.9 (<https://github.com/photocyte/mzmine2/commit/609bb3b1811b3df4ff632100591a2e449564eaf2>). The change has been contributed to the main MZmine2 sourcecode repository.

13. The resulting peaklist without annotated adducts, complexes, and in-source fragment ions was exported as “spermatophore_vs_body_final_filtered.mzTab” (Supplementary MetaboLights Data - MTBLS362)

14. Resulting compounds were manually annotated using a combination of the “Online database search” module & “Formula prediction” module. When possible, MS^2^ spectra were compared with the Metlin metabolite database (metlin.scripps.edu)

**Supplementary Figure S1**. Biological coefficient of variation analysis of transcript abundance (normalized read counts) in male *P. pyralis* spiral accessory glands (SpAGs), other accessory glands (OAGs), fat body, male thorax, female spermatophore digesting gland and bursa copulatrix (B) spermatheca, and female thorax. The X- and Y-axes approximate the expression differences between samples using LogFC.


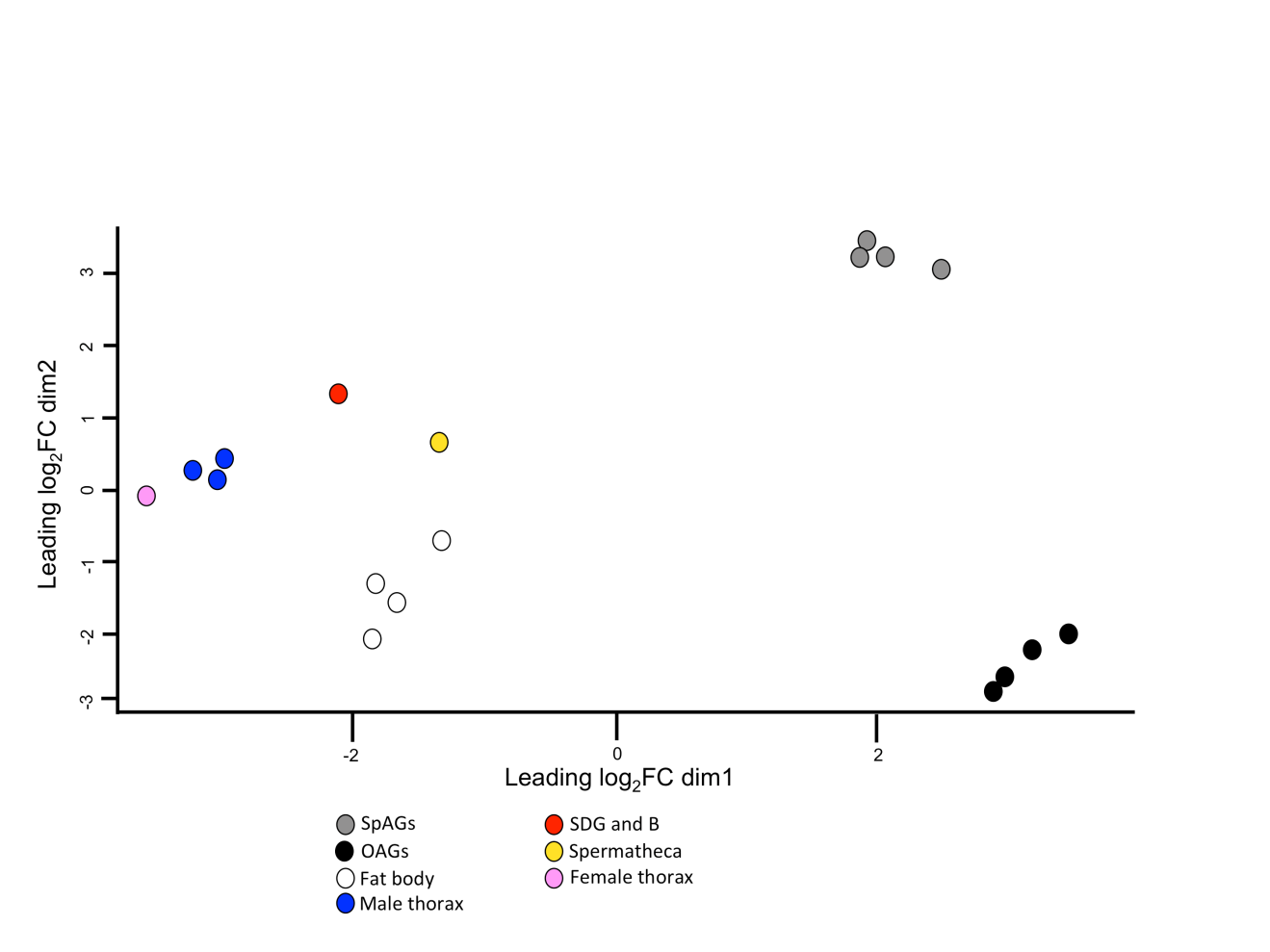


**Supplementary Figure S2**. Planned comparisons between *P. pyralis* firefly male and female tissues for differential expression analysis (reproductive tissues are enclosed within dashed lines). Two-way arrows indicate that both up- and down-regulated genes were examined, while one-way arrows indicate that only genes up-regulated in the destination tissue were examined (e.g. genes up-regulated in male accessory glands compared to male thorax).


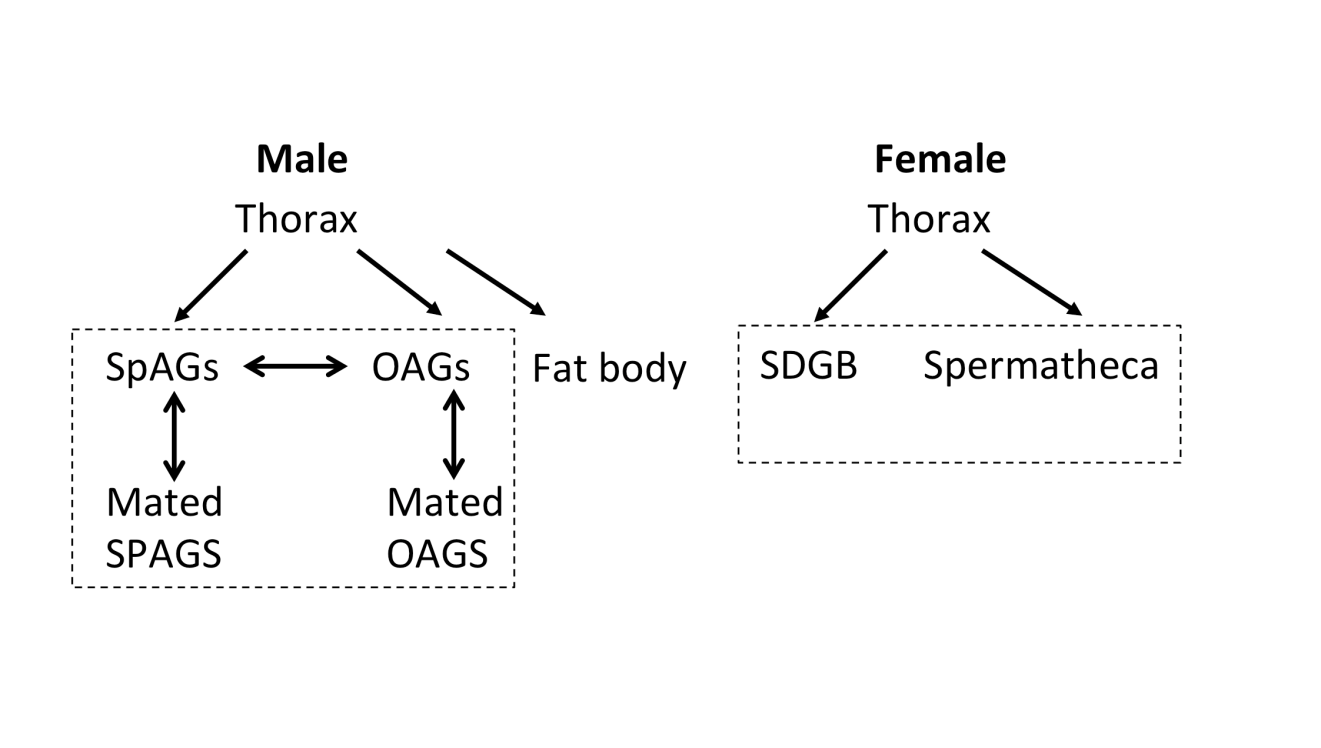


**Supplementary Figure S3**: C18 LC-HRAM-MS base peak chromatogram of a methanolic extract of a single whole male *P. pyralis* firefly with the last two abdominal segments removed (body).

| **Tissue comparison** | **Number of DE Sequences** | **Sequences Containing Transmembrane helices (n)** | **Sequences Containing Secretion signal (n)** | **Annotated sequences (%, n)** |
| --- | --- | --- | --- | --- |
| Male SpAGs  *vs* Thorax* | 1891 | 362 | 206 | 30%, 573 |
| Male OAGs *vs* Thorax* | 1403 | 509 | 253 | 40%, 562 |
| Male OAGs *vs* Mated Male OAG† | 26 | 0 | 0 | 50%, 13 |
| Male SpAGs *vs*  Mated Male SpAGs† | 47 | 0 | 0 | 43%, 20 |

**Supplementary Table 1**. Summary statistics for annotated sequences that were differentially expressed in *P. pyralis* male reproductive tissues.

* Statistics presented are for genes up-regulated in reproductive tissue compared to thorax

† Statistics presented are for genes up-regulated in tissues dissected from recently mated males compared to males with unknown mating status

**Supplementary Table 2**. Sequences that were differentially expressed between spiral accessory glands (SpAGs) and other accessory glands (OAGs).

| **Tissue** | | **Sequence ID** | **Sequence description** | **Log FC** |
| --- | --- | --- | --- | --- |
| **Up-regulated in SpAGs** | | | | |
|  | 151_Ppyr_v3_TRINITY_DN15036_c0_g1_i8 | | ADAMTs | -11.8 |
|  | 151_Ppyr_v3_TRINITY_DN14534_c0_g1_i2 | | arrestin | -11.1 |
|  | 151_Ppyr_v3_TRINITY_DN16148_c0_g1_i4 | | mitochondrial sodium hydrogen exchanger | -10.9 |
|  | 151_Ppyr_v3_TRINITY_DN16096_c0_g2_i1 | | slit homolog 2 | -9.1 |
|  | 151_Ppyr_v3_TRINITY_DN11011_c0_g1_i1 | | amino acid transporter 2 | -9 |
|  | 151_Ppyr_v3_TRINITY_DN12177_c0_g1_i2 | | early growth response | -7.8 |
|  | 151_Ppyr_v3_TRINITY_DN12177_c0_g1_i1 | | early growth response | -7.6 |
|  | 151_Ppyr_v3_TRINITY_DN10656_c0_g1_i1 | | growth arrest-specific 1 | -7.6 |
|  | 151_Ppyr_v3_TRINITY_DN9179_c0_g1_i1 | | Arylsulfatase B | -7 |
|  | 151_Ppyr_v3_TRINITY_DN16946_c0_g1_i1 | | pancreatic triacylglycerol lipase | -6.1 |
|  | 151_Ppyr_v3_TRINITY_DN14209_c1_g1_i1 | | calmodulin | -4.7 |
|  | 151_Ppyr_v3_TRINITY_DN8820_c0_g1_i2 | | calcium-transporting ATPase | -2.6 |
|  | 151_Ppyr_v3_TRINITY_DN9701_c0_g1_i1 | | HLH domain containing protein | -2.1 |
| **Up-regulated in OAGs** | | | | |
|  | 151_Ppyr_v3_TRINITY_DN15353_c0_g1_i1 | | major facilitator superfamily domain-containing protein 11 | 4.6 |
|  | 151_Ppyr_v3_TRINITY_DN9708_c0_g1_i1 | | glyco 3-alpha-L-fucosyltransferase A | 5.1 |
|  | 151_Ppyr_v3_TRINITY_DN16194_c0_g1_i1 | | G-activated inward rectifier potassium channel | 5.2 |
|  | 151_Ppyr_v3_TRINITY_DN12677_c0_g1_i1 | | prostaglandin reductase 1 | 5.3 |
|  | 151_Ppyr_v3_TRINITY_DN16994_c0_g1_i3 | | monocarboxylate transporter 14 | 5.9 |
|  | 151_Ppyr_v3_TRINITY_DN9302_c0_g1_i1 | | facilitated trehalose transporter 1 | 7.8 |
|  | 151_Ppyr_v3_TRINITY_DN2181_c0_g1_i1 | | myrosinase 1 | 8.2 |
|  | 151_Ppyr_v3_TRINITY_DN14805_c0_g6_i3 | | barrier-to-autointegration factor | 8.2 |
|  | 151_Ppyr_v3_TRINITY_DN24212_c0_g2_i1 | | ATP-sensitive inward rectifier potassium channel | 8.4 |
|  | 151_Ppyr_v3_TRINITY_DN9136_c0_g2_i1 | | sodium dependent phosphate transporter | 8.5 |
|  | 151_Ppyr_v3_TRINITY_DN13488_c0_g1_i1 | | apyrase | 8.7 |
|  | 151_Ppyr_v3_TRINITY_DN12471_c0_g1_i1 | | Leucine-rich repeat and WD reapeat containing protein | 8.8 |
|  | 151_Ppyr_v3_TRINITY_DN9507_c0_g1_i1 | | homeobox araucan | 11.6 |
|  | 151_Ppyr_v3_TRINITY_DN10251_c0_g1_i2 | | glycine N-acyltransferase | 12.8 |

**Supplementary Table S3**. Proposed male tissues of *P. pyralis* fireflies for transcripts encoding spermatophore proteins that could not be annotated.

| **Tissue** and protein functional class | Sequence ID | MW (kDa) | Gel Section | Predicted Signal Peptide? |
| --- | --- | --- | --- | --- |
| **Spiral Accessory Glands (SpAGs)** | | | | |
| Novel | | | | |
|  | 151_Ppyr_v3_TRINITY_DN1435_c3_g5_i1 | 185 | 1 |  |
|  | 151_Ppyr_v3_TRINITY_DN1435_c3_g6_i1 | 14 | 7 |  |
|  | 151_Ppyr_v3_TRINITY_DN1435_c3_g8_i1 | 239 | 1 |  |
|  | 151_Ppyr_v3_TRINITY_DN5809_c1_g1_i1 | 35 | 4 | + |
|  | 151_Ppyr_v3_TRINITY_DN5981_c0_g1_i1 | 13 | 7 |  |
|  | 151_Ppyr_v3_TRINITY_DN16695_c0_g1_i1 | 56 | 3 |  |
|  | 151_Ppyr_v3_TRINITY_DN2808_c0_g1_i1 | 13 | 7 |  |
|  | 151_Ppyr_v3_TRINITY_DN5177_c0_g1_i1 | 14 | 7 | + |
|  | 151_Ppyr_v3_TRINITY_DN6688_c0_g1_i1 | 13 | 7 | + |
|  | 151_Ppyr_v3_TRINITY_DN12693_c3_g3_i1 | 71 | 3 |  |
|  | 151_Ppyr_v3_TRINITY_DN15103_c0_g2_i1 | 49 | 4 |  |
|  | 151_Ppyr_v3_TRINITY_DN14054_c0_g1_i1 | 82 | 2 | + |
|  | 151_Ppyr_v3_TRINITY_DN15103_c0_g1_i1 | 15 | 7 |  |
|  | 151_Ppyr_v3_TRINITY_DN4568_c0_g1_i1 | 20 | 6 |  |
|  | 151_Ppyr_v3_TRINITY_DN17631_c0_g1_i2 | 250 | 1 |  |
|  | 151_Ppyr_v3_TRINITY_DN10347_c0_g1_i1 | 28 | 5 | + |
| **Other Accessory Glands (OAGs)** | | | | |
| Novel | | | | |
|  | 151_Ppyr_v3_TRINITY_DN30894_c2_g1_i1 | 30 | 5 |  |
|  | 151_Ppyr_v3_TRINITY_DN8691_c0_g1_i1 | 61 | 3 | + |
|  | 151_Ppyr_v3_TRINITY_DN17778_c0_g2_i2 | 63 | 3 |  |
|  | 151_Ppyr_v3_TRINITY_DN17778_c0_g2_i1 | 44 | 4 |  |
|  | 151_Ppyr_v3_TRINITY_DN14800_c0_g1_i1 | 69 | 3 | + |
|  | 151_Ppyr_v3_TRINITY_DN13010_c0_g1_i4 | 44 | 4 | + |
|  | 151_Ppyr_v3_TRINITY_DN6834_c0_g1_i2 | 35 | 5 | + |
|  | 151_Ppyr_v3_TRINITY_DN17799_c6_g3_i11 | 50 | 4 |  |
|  | 151_Ppyr_v3_TRINITY_DN15764_c0_g1_i1 | 48 | 4 | + |
|  | 151_Ppyr_v3_TRINITY_DN10439_c0_g1_i1 | 15 | 7 | + |
|  | 151_Ppyr_v3_TRINITY_DN2338_c0_g1_i1 | 50 | 4 |  |
|  | 151_Ppyr_v3_TRINITY_DN13548_c0_g2_i1 | 45 | 4 | + |
|  | 151_Ppyr_v3_TRINITY_DN12812_c0_g1_i1 | 23 | 6 | + |
|  | 151_Ppyr_v3_TRINITY_DN16243_c0_g1_i1 | 12 | 7 | + |
|  | 151_Ppyr_v3_TRINITY_DN17761_c0_g1_i1 | 13 | 7 |  |
|  | 151_Ppyr_v3_TRINITY_DN2953_c0_g2_i1 | 20 | 6 | + |
|  | 151_Ppyr_v3_TRINITY_DN16752_c1_g1_i1 | 82 | 2 |  |
|  | 151_Ppyr_v3_TRINITY_DN6760_c0_g1_i1 | 53 | 4 | + |
|  | 151_Ppyr_v3_TRINITY_DN2319_c1_g1_i1 | 11 | 8 | + |
|  | 151_Ppyr_v3_TRINITY_DN14594_c0_g1_i1 | 83 | 2 |  |
|  | 151_Ppyr_v3_TRINITY_DN17799_c6_g3_i7 | 22 | 6 |  |
| **Fat body** | | | | |
| Novel | | | | |
|  | 151_Ppyr_v3_TRINITY_DN13548_c0_g2_i1 | 45 | 4 | + |
|  | 151_Ppyr_v3_TRINITY_DN5991_c0_g1_i1 | 43 | 4 |  |
|  | 151_Ppyr_v3_TRINITY_DN12416_c3_g1_i2 | 370 | 1 | + |
|  | 151_Ppyr_v3_TRINITY_DN17128_c1_g1_i1 | 100 | 2 | + |
|  | 151_Ppyr_v3_TRINITY_DN17195_c0_g2_i1 | 24 | 5 | + |
|  | 151_Ppyr_v3_TRINITY_DN6374_c0_g1_i1 | 74 | 3 | + |

**Supplementary Table S4**: Annotations for additional transcripts encoding proteins identified in the *P. pyralis* male spermatophore.

| **Accession Number** | **Description** | **Signal peptide?** |
| --- | --- | --- |
| **Antimicrobial and immune response proteins** | |  |
| 151_Ppyr_v3_TRINITY_DN12083_c0_g1_i1 | phenoloxidase |  |
| 151_Ppyr_v3_TRINITY_DN16010_c0_g1_i1 | phenoloxidase |  |
| 151_Ppyr_v3_TRINITY_DN61042_c0_g1_i1 | phenoloxidase |  |
| 151_Ppyr_v3_TRINITY_DN36635_c0_g1_i1 | phenoloxidase |  |
| **Carbohydrate metabolic process** |  |  |
| 151_Ppyr_v3_TRINITY_DN15451_c0_g1_i1 | chitooligosaccharidolytic beta-N-acetylglucosaminidase | + |
| 151_Ppyr_v3_TRINITY_DN14087_c0_g2_i1 | chitinase EN03 |  |
| 151_Ppyr_v3_TRINITY_DN6995_c0_g1_i1 | Neutral alpha-glucosidase AB | + |
| **Signal transduction** |  |  |
| 151_Ppyr_v3_TRINITY_DN12946_c0_g1_i1 | 14-3-3 epsilon |  |
| **Peptidases and peptidase regulators** |  |  |
| 151_Ppyr_v3_TRINITY_DN9669_c0_g1_i1 | serine protease |  |
| 151_Ppyr_v3_TRINITY_DN10938_c0_g1_i1 | serine protease snake | + |
| 151_Ppyr_v3_TRINITY_DN8730_c0_g1_i1 | transmembrane protease serine 9 | + |
| 151_Ppyr_v3_TRINITY_DN5988_c0_g1_i1 | angiotensin-converting enzyme | + |
| 151_Ppyr_v3_TRINITY_DN15769_c1_g1_i1 | aminopeptidase N | + |
| 151_Ppyr_v3_TRINITY_DN17149_c1_g1_i1 | aminopeptidase N | + |
| 151_Ppyr_v3_TRINITY_DN10619_c0_g1_i1 | carboxypeptidase Q | + |
| 151_Ppyr_v3_TRINITY_DN16325_c0_g2_i1 | Cathepsin L | + |
| 151_Ppyr_v3_TRINITY_DN4855_c0_g1_i1 | Cathepsin L | + |
| 151_Ppyr_v3_TRINITY_DN16477_c0_g1_i1 | cysteine ase CG12163 |  |
| 151_Ppyr_v3_TRINITY_DN10232_c0_g1_i1 | digestive cysteine ase 1 | + |
| 151_Ppyr_v3_TRINITY_DN10909_c0_g1_i1 | Serine protease easter | + |
| 151_Ppyr_v3_TRINITY_DN13786_c0_g1_i1 | Serine ase stubble | + |
| 151_Ppyr_v3_TRINITY_DN16168_c0_g1_i1 | gamma-glutamyltranspeptidase 1 |  |
| 151_Ppyr_v3_TRINITY_DN10673_c0_g1_i1 | lysosomal aspartic protease | + |
| 151_Ppyr_v3_TRINITY_DN14826_c0_g1_i1 | membrane metallo-endopeptidase 1 |  |
| 151_Ppyr_v3_TRINITY_DN12753_c1_g1_i4 | membrane metallo-endopeptidase 1 |  |
| **Development** |  |  |
| 151_Ppyr_v3_TRINITY_DN9304_c0_g1_i1 | transcription factor ken | + |
| **Structural** |  |  |
| 151_Ppyr_v3_TRINITY_DN4899_c0_g1_i1 | actin |  |
| 151_Ppyr_v3_TRINITY_DN6767_c0_g1_i2 | annexin B9 |  |
| 151_Ppyr_v3_TRINITY_DN11298_c0_g1_i1 | annexin B10 |  |
| 151_Ppyr_v3_TRINITY_DN40252_c0_g1_i1 | Tubulin beta chain |  |
| 151_Ppyr_v3_TRINITY_DN4678_c0_g1_i1 | Tubulin beta chain |  |
| 151_Ppyr_v3_TRINITY_DN11630_c0_g1_i4 | troponin T |  |
| 151_Ppyr_v3_TRINITY_DN11348_c0_g1_i10 | troponin I |  |
| 151_Ppyr_v3_TRINITY_DN11348_c0_g1_i11 | troponin I |  |
| 151_Ppyr_v3_TRINITY_DN8862_c0_g1_i1 | tubulin alpha-1 |  |
| 151_Ppyr_v3_TRINITY_DN41067_c0_g1_i1 | tubulin alpha-1C | + |
| 151_Ppyr_v3_TRINITY_DN15129_c0_g1_i2 | spectrin alpha chain |  |
| 151_Ppyr_v3_TRINITY_DN7496_c0_g1_i1 | troponin C |  |
| 151_Ppyr_v3_TRINITY_DN12255_c0_g1_i1 | cuticular RR-2 | + |
| 151_Ppyr_v3_TRINITY_DN2226_c0_g1_i1 | cofilin actin-depolymerizing factor homolog |  |
| 151_Ppyr_v3_TRINITY_DN1828_c0_g1_i1 | endocuticle structural glyco bd-3 | + |
| 151_Ppyr_v3_TRINITY_DN11462_c0_g1_i1 | Tubulin beta-1 |  |
| 151_Ppyr_v3_TRINITY_DN14107_c0_g1_i1 | Tubulin alpha-1 |  |
| 151_Ppyr_v3_TRINITY_DN10944_c2_g1_i1 | Spectrin beta chain |  |
| 151_Ppyr_v3_TRINITY_DN5992_c0_g1_i1 | myosin regulatory light chain 2 |  |
| 151_Ppyr_v3_TRINITY_DN6933_c0_g1_i1 | myosin light chain alkali |  |
| 151_Ppyr_v3_TRINITY_DN8786_c0_g1_i1 | myophilin |  |
| 151_Ppyr_v3_TRINITY_DN7522_c0_g1_i1 | myophilin |  |
| 151_Ppyr_v3_TRINITY_DN11793_c0_g1_i1 | muscle-specific 20 |  |
| 151_Ppyr_v3_TRINITY_DN8157_c0_g1_i2 | muscle LIM MIp84B |  |
| 151_Ppyr_v3_TRINITY_DN48792_c0_g1_i1 | muscle |  |
| 151_Ppyr_v3_TRINITY_DN14440_c0_g1_i1 | glycogen phosphorylase |  |
| 151_Ppyr_v3_TRINITY_DN19550_c0_g1_i1 | glycine-rich cell wall structure | + |
| 151_Ppyr_v3_TRINITY_DN17477_c0_g1_i1 | Laminin subunit gamma-1 | + |
| 151_Ppyr_v3_TRINITY_DN17711_c0_g1_i3 | PDZ and LIM domain Zasp |  |
| 151_Ppyr_v3_TRINITY_DN12682_c2_g1_i1 | paxillin |  |
| 151_Ppyr_v3_TRINITY_DN5860_c0_g1_i1 | profilin |  |
| 151_Ppyr_v3_TRINITY_DN55009_c0_g1_i1 | pupal cuticle |  |
| **General cellular processes** |  |  |
| 151_Ppyr_v3_TRINITY_DN8346_c0_g1_i1 | von Willebrand factor D and EGF domain-containing |  |
| 151_Ppyr_v3_TRINITY_DN5244_c0_g1_i1 | vacuolar sorting-associated 52 |  |
| 151_Ppyr_v3_TRINITY_DN6721_c0_g1_i1 | ribosomal L14 |  |
| 151_Ppyr_v3_TRINITY_DN22312_c0_g1_i1 | ubiquitin-conjugating enzyme E2 |  |
| 151_Ppyr_v3_TRINITY_DN9650_c0_g1_i1 | transitional endoplasmic reticulum ATPase |  |
| 151_Ppyr_v3_TRINITY_DN2260_c0_g1_i1 | ubiquitin carboxyl-terminal hydrolase isozyme L3 |  |
| 151_Ppyr_v3_TRINITY_DN16145_c0_g1_i1 | trifunctional enzyme |  |
| 151_Ppyr_v3_TRINITY_DN7599_c0_g1_i1 | succinate dehydrogenase |  |
| 151_Ppyr_v3_TRINITY_DN22141_c0_g1_i1 | superoxide dismutase |  |
| 151_Ppyr_v3_TRINITY_DN14481_c1_g1_i1 | superoxide dismutase | + |
| 151_Ppyr_v3_TRINITY_DN12323_c0_g1_i1 | triosephosphate isomerase |  |
| 151_Ppyr_v3_TRINITY_DN9674_c0_g1_i1 | 60S ribosomal L9 |  |
| 151_Ppyr_v3_TRINITY_DN48836_c0_g1_i1 | 60S ribosomal L31 |  |
| 151_Ppyr_v3_TRINITY_DN22126_c0_g1_i1 | 60S ribosomal L23 |  |
| 151_Ppyr_v3_TRINITY_DN13259_c0_g1_i1 | 60 kDa heat shock mitochondrial |  |
| 151_Ppyr_v3_TRINITY_DN12961_c1_g1_i2 | 6-phosphofructo-2-kinase fructose-2,6-bisphosphatase 1 |  |
| 151_Ppyr_v3_TRINITY_DN12873_c0_g1_i2 | 40S ribosomal S9 |  |
| 151_Ppyr_v3_TRINITY_DN10001_c0_g1_i1 | 40S ribosomal S5 |  |
| 151_Ppyr_v3_TRINITY_DN1419_c0_g1_i1 | 40S ribosomal S13 |  |
| 151_Ppyr_v3_TRINITY_DN58249_c0_g1_i1 | 40S ribosomal S10-like |  |
| 151_Ppyr_v3_TRINITY_DN22108_c0_g1_i1 | 4-aminobutyrate mitochondrial |  |
| 151_Ppyr_v3_TRINITY_DN8639_c0_g1_i1 | 39S ribosomal mitochondrial |  |
| 151_Ppyr_v3_TRINITY_DN7521_c0_g1_i1 | 14-3-3 zeta isoform X1 |  |
| 151_Ppyr_v3_TRINITY_DN7521_c0_g1_i3 | 14-3-3 zeta isoform X1 |  |
| 151_Ppyr_v3_TRINITY_DN14336_c0_g1_i1 | alpha-sarcomeric |  |
| 151_Ppyr_v3_TRINITY_DN12220_c0_g1_i1 | alpha-N-acetylgalactosaminidase | + |
| 151_Ppyr_v3_TRINITY_DN10122_c0_g2_i1 | alpha-L-fucosidase |  |
| 151_Ppyr_v3_TRINITY_DN15260_c0_g1_i1 | adenylate kinase isoenzyme 1 isoform |  |
| 151_Ppyr_v3_TRINITY_DN53061_c0_g1_i1 | adenylate kinase isoenzyme 1 isoform |  |
| 151_Ppyr_v3_TRINITY_DN7600_c0_g1_i1 | adenine phosphoribosyltranferase | + |
| 151_Ppyr_v3_TRINITY_DN7470_c1_g1_i1 | Beta-1,3-galactotransferase brn |  |
| 151_Ppyr_v3_TRINITY_DN40076_c0_g1_i1 | ATP synthase subunit mitochondrial |  |
| 151_Ppyr_v3_TRINITY_DN13536_c0_g1_i1 | ATP synthase subunit mitochondrial |  |
| 151_Ppyr_v3_TRINITY_DN10157_c0_g1_i1 | ATP synthase subunit mitochondrial |  |
| 151_Ppyr_v3_TRINITY_DN1753_c0_g1_i1 | ATP synthase subunit mitochondrial |  |
| 151_Ppyr_v3_TRINITY_DN1656_c0_g1_i1 | ATP synthase subunit |  |
| 151_Ppyr_v3_TRINITY_DN10376_c0_g1_i1 | ATP carrier |  |
| 151_Ppyr_v3_TRINITY_DN34519_c0_g1_i1 | ATP carrier |  |
| 151_Ppyr_v3_TRINITY_DN11783_c0_g1_i1 | aspartate mitochondrial |  |
| 151_Ppyr_v3_TRINITY_DN7056_c1_g1_i1 | arylsulfatase B | + |
| 151_Ppyr_v3_TRINITY_DN8849_c0_g1_i1 | Calmodulin |  |
| 151_Ppyr_v3_TRINITY_DN7080_c0_g1_i1 | C-type lectin isoform X1 | + |
| 151_Ppyr_v3_TRINITY_DN15349_c0_g1_i1 | beta-glucuronidase | + |
| 151_Ppyr_v3_TRINITY_DN15562_c0_g1_i1 | beta-galactosidase-1 2 |  |
| 151_Ppyr_v3_TRINITY_DN4074_c0_g2_i1 | Catalase |  |
| 151_Ppyr_v3_TRINITY_DN50812_c0_g1_i1 | Catalase |  |
| 151_Ppyr_v3_TRINITY_DN6187_c0_g1_i1 | CG31997 | + |
| 151_Ppyr_v3_TRINITY_DN42963_c0_g1_i1 | CDGSH iron-sulfur domain-containing 2 |  |
| 151_Ppyr_v3_TRINITY_DN5028_c0_g1_i1 | cdc42 |  |
| 151_Ppyr_v3_TRINITY_DN6022_c0_g1_i1 | cytosolic none-specific dipeptidase |  |
| 151_Ppyr_v3_TRINITY_DN10917_c0_g1_i1 | cytoplasmic NADP-dependent isocitrate |  |
| 151_Ppyr_v3_TRINITY_DN23117_c0_g1_i1 | cytoplasmic 1 |  |
| 151_Ppyr_v3_TRINITY_DN4792_c0_g1_i1 | cytochrome heme |  |
| 151_Ppyr_v3_TRINITY_DN4994_c0_g1_i1 | cytochrome c-2 |  |
| 151_Ppyr_v3_TRINITY_DN58646_c0_g1_i1 | cytochrome c oxidase |  |
| 151_Ppyr_v3_TRINITY_DN12285_c0_g1_i1 | Cytochrome b-c1 complex subunit mitochondrial |  |
| 151_Ppyr_v3_TRINITY_DN32056_c0_g1_i1 | cytochrome b-c1 complex |  |
| 151_Ppyr_v3_TRINITY_DN13818_c0_g1_i1 | delta-1-pyrroline-5-carboxylate |  |
| 151_Ppyr_v3_TRINITY_DN13560_c0_g1_i1 | D-arabinitol dehydrogenase 1 |  |
| 151_Ppyr_v3_TRINITY_DN13270_c0_g1_i1 | Volatage-dependent anion-selective channel |  |
| 151_Ppyr_v3_TRINITY_DN6818_c0_g1_i1 | V-type proton ATPase catalytic subunit S1 | + |
| 151_Ppyr_v3_TRINITY_DN1632_c0_g1_i1 | V-type proton ATPase catalytic subunit G |  |
| 151_Ppyr_v3_TRINITY_DN58678_c0_g1_i1 | V-type proton ATPase catalytic subunit F |  |
| 151_Ppyr_v3_TRINITY_DN2233_c1_g1_i1 | V-type proton ATPase catalytic subunit E |  |
| 151_Ppyr_v3_TRINITY_DN10946_c0_g1_i1 | V-type proton ATPase catalytic subunit D1 |  |
| 151_Ppyr_v3_TRINITY_DN14413_c0_g1_i1 | V-type proton ATPase catalytic subunit C |  |
| 151_Ppyr_v3_TRINITY_DN1410_c0_g1_i1 | V-type proton ATPase catalytic subunit B |  |
| 151_Ppyr_v3_TRINITY_DN13283_c0_g1_i1 | V-type proton ATPase catalytic subunit A |  |
| 151_Ppyr_v3_TRINITY_DN2178_c0_g1_i1 | UMP-CMP kinase |  |
| 151_Ppyr_v3_TRINITY_DN8371_c0_g1_i1 | elongation factor 1 |  |
| 151_Ppyr_v3_TRINITY_DN27341_c0_g1_i1 | elongation factor 1 |  |
| 151_Ppyr_v3_TRINITY_DN54_c0_g1_i1 | disulfide-isomerase A3 | + |
| 151_Ppyr_v3_TRINITY_DN15467_c0_g2_i1 | dihydrolipoyllysine-reside acetyltransferase |  |
| 151_Ppyr_v3_TRINITY_DN8547_c0_g1_i1 | dihydrolipoamide dehydrogenase E3 |  |
| 151_Ppyr_v3_TRINITY_DN13943_c0_g1_i1 | eukaryotic translation initiation factor 5A |  |
| 151_Ppyr_v3_TRINITY_DN49586_c0_g1_i1 | eukaryotic translation initiation factor 2 Y-linked |  |
| 151_Ppyr_v3_TRINITY_DN5489_c0_g1_i1 | enolase |  |
| 151_Ppyr_v3_TRINITY_DN12567_c0_g1_i1 | fructose-bisphosphatase aldolase |  |
| 151_Ppyr_v3_TRINITY_DN14233_c0_g1_i1 | fructose-bisphosphatase aldolase |  |
| 151_Ppyr_v3_TRINITY_DN11409_c0_g1_i1 | farnesoic acid O-methyltransferase |  |
| 151_Ppyr_v3_TRINITY_DN11409_c0_g2_i1 | farnesoic acid O-methyltransferase |  |
| 151_Ppyr_v3_TRINITY_DN1067_c0_g1_i1 | farnesoic acid O-methyltransferase |  |
| 151_Ppyr_v3_TRINITY_DN10755_c0_g1_i1 | NADP-dependent malic enzyme |  |
| 151_Ppyr_v3_TRINITY_DN5182_c0_g1_i1 | NADH-ubiquinone oxidoreductase subunit 8 |  |
| 151_Ppyr_v3_TRINITY_DN14301_c0_g1_i1 | NADH dehydrogenase iron-sulfur |  |
| 151_Ppyr_v3_TRINITY_DN11379_c0_g1_i1 | NADH dehydrogenase 1 |  |
| 151_Ppyr_v3_TRINITY_DN22415_c0_g1_i1 | NADH dehydrogenase 1 |  |
| 151_Ppyr_v3_TRINITY_DN1514_c0_g1_i1 | NADH dehydrogenase 1 |  |
| 151_Ppyr_v3_TRINITY_DN5919_c0_g1_i1 | NADH dehydrogenase 1 |  |
| 151_Ppyr_v3_TRINITY_DN10193_c0_g1_i1 | NADH dehydrogenase 1 |  |
| 151_Ppyr_v3_TRINITY_DN9022_c0_g1_i1 | glyceraldehyde-3-phosphate dehydrogenase |  |
| 151_Ppyr_v3_TRINITY_DN7572_c0_g1_i1 | Glutathione S-transferase |  |
| 151_Ppyr_v3_TRINITY_DN16493_c0_g1_i1 | Glutamate mitochondrial |  |
| 151_Ppyr_v3_TRINITY_DN11197_c1_g1_i1 | histone H4 |  |
| 151_Ppyr_v3_TRINITY_DN11025_c0_g1_i1 | histone H2B |  |
| 151_Ppyr_v3_TRINITY_DN1631_c0_g1_i1 | high mobility group DSP 1 |  |
| 151_Ppyr_v3_TRINITY_DN17199_c2_g1_i1 | Hermansky-Pudlak syndrome 4 |  |
| 151_Ppyr_v3_TRINITY_DN15081_c0_g1_i1 | heat shock 70 kDa cognate 3 | + |
| 151_Ppyr_v3_TRINITY_DN12693_c3_g3_i1 | heat shock 70 |  |
| 151_Ppyr_v3_TRINITY_DN22898_c0_g1_i1 | heat shock 70 |  |
| 151_Ppyr_v3_TRINITY_DN22911_c0_g1_i1 | heat shock 1 |  |
| 151_Ppyr_v3_TRINITY_DN5987_c0_g1_i1 | guanine deaminase | + |
| 151_Ppyr_v3_TRINITY_DN7428_c0_g1_i1 | GTP-binding SAR1b |  |
| 151_Ppyr_v3_TRINITY_DN8247_c0_g1_i1 | proliferating cell nuclear antigen |  |
| 151_Ppyr_v3_TRINITY_DN19462_c0_g1_i1 | Programmed cell death 6 |  |
| 151_Ppyr_v3_TRINITY_DN9439_c0_g1_i1 | Programmed cell death 6 |  |
| 151_Ppyr_v3_TRINITY_DN11875_c0_g1_i1 | peroxiredoxin |  |
| 151_Ppyr_v3_TRINITY_DN58267_c0_g1_i1 | peptidyl-prolyl cis-trans isomerase 5 | + |
| 151_Ppyr_v3_TRINITY_DN7024_c0_g1_i1 | peptidyl-prolyl cis-trans isomerase |  |
| 151_Ppyr_v3_TRINITY_DN12493_c0_g1_i2 | peptidoglycan-recognition LB |  |
| 151_Ppyr_v3_TRINITY_DN14214_c0_g1_i1 | lamin Dm0 |  |
| 151_Ppyr_v3_TRINITY_DN53411_c0_g1_i1 | lamin Dm0 |  |
| 151_Ppyr_v3_TRINITY_DN48962_c0_g1_i1 | isocitrate dehydrogenase |  |
| 151_Ppyr_v3_TRINITY_DN1856_c0_g1_i1 | integument esterase | + |
| 151_Ppyr_v3_TRINITY_DN17778_c0_g2_i6 | insulin-like growth factor-binding complex |  |
| 151_Ppyr_v3_TRINITY_DN9981_c0_g1_i1 | RNA-binding squid |  |
| 151_Ppyr_v3_TRINITY_DN10620_c0_g1_i1 | ras-related Rac1 |  |
| 151_Ppyr_v3_TRINITY_DN5313_c0_g1_i1 | ras-like GTP-binding Rho 1 |  |
| 151_Ppyr_v3_TRINITY_DN8233_c1_g1_i1 | Pyruvate kinase |  |
| 151_Ppyr_v3_TRINITY_DN22331_c0_g1_i1 | probable enoyl-mitochondrial |  |
| 151_Ppyr_v3_TRINITY_DN13079_c0_g1_i1 | probable citrate synthase |  |
| 151_Ppyr_v3_TRINITY_DN13421_c0_g1_i1 | polyubiquitin-B |  |
| 151_Ppyr_v3_TRINITY_DN14322_c0_g1_i6 | phospholipid hydroperoxide glutathione peroxidase | + |
| 151_Ppyr_v3_TRINITY_DN14322_c0_g1_i5 | phospholipid hydroperoxide glutathione peroxidase |  |
| 151_Ppyr_v3_TRINITY_DN40713_c0_g1_i1 | phosphoglycerate mutase 1 |  |
| 151_Ppyr_v3_TRINITY_DN14350_c0_g1_i1 | phosphoglycerate kinase |  |
| 151_Ppyr_v3_TRINITY_DN6698_c0_g1_i1 | Nucleoside diphosphatase kinase |  |
| 151_Ppyr_v3_TRINITY_DN8274_c0_g1_i1 | Nucleoside diphosphatase kinase |  |
| 151_Ppyr_v3_TRINITY_DN9600_c0_g1_i1 | Mitochondrial-processing peptidase subunit beta |  |
| 151_Ppyr_v3_TRINITY_DN10304_c0_g1_i1 | mitochondrial hydriogen-transporting ATP synthase |  |
| 151_Ppyr_v3_TRINITY_DN9630_c3_g1_i1 | mitochondrial enolase superfamily member 1 |  |
| 151_Ppyr_v3_TRINITY_DN1525_c0_g1_i1 | microsomal glutathione S-transferase 1 |  |
| 151_Ppyr_v3_TRINITY_DN1238_c0_g1_i1 | MICOS complex subunit MIC13 homolog |  |
| 151_Ppyr_v3_TRINITY_DN1766_c0_g1_i1 | methylmalonate-semialdehyde dehydrogenase |  |
| 151_Ppyr_v3_TRINITY_DN10178_c0_g1_i1 | malate mitochondrial |  |
| 151_Ppyr_v3_TRINITY_DN58181_c0_g1_i1 | malate cytoplasmic |  |
| 151_Ppyr_v3_TRINITY_DN15632_c0_g1_i2 | long form |  |
| 151_Ppyr_v3_TRINITY_DN10972_c0_g1_i1 | leucine-rich repeat-containing G-coupled receptor 4 |  |
| **Conserved unknown proteins** |  |  |
| 151_Ppyr_v3_TRINITY_DN13942_c0_g1_i1 | uncharacterized protein LOC656585 | + |
| **Novel** |  |  |
| 151_Ppyr_v3_TRINITY_DN10347_c0_g1_i1 | novel |  |
| 151_Ppyr_v3_TRINITY_DN17195_c0_g1_i1 | novel |  |
| 151_Ppyr_v3_TRINITY_DN10169_c0_g1_i2 | novel |  |
| 151_Ppyr_v3_TRINITY_DN6834_c0_g1_i2 | novel | + |
| 151_Ppyr_v3_TRINITY_DN11348_c0_g1_i3 | novel |  |
| 151_Ppyr_v3_TRINITY_DN13548_c0_g2_i1 | novel | + |
| 151_Ppyr_v3_TRINITY_DN17143_c2_g1_i3 | novel |  |
| 151_Ppyr_v3_TRINITY_DN13384_c0_g1_i1 | novel | + |
| 151_Ppyr_v3_TRINITY_DN15712_c0_g1_i3 | novel | + |
| 151_Ppyr_v3_TRINITY_DN12812_c0_g1_i1 | novel | + |
| 151_Ppyr_v3_TRINITY_DN5986_c0_g1_i1 | novel | + |
| 151_Ppyr_v3_TRINITY_DN48816_c0_g1_i1 | novel |  |
| 151_Ppyr_v3_TRINITY_DN17195_c0_g2_i1 | novel | + |
| 151_Ppyr_v3_TRINITY_DN5042_c0_g1_i1 | novel |  |
| 151_Ppyr_v3_TRINITY_DN6760_c0_g1_i1 | novel | + |
| 151_Ppyr_v3_TRINITY_DN22490_c0_g1_i1 | novel | + |
| 151_Ppyr_v3_TRINITY_DN16358_c0_g1_i1 | novel | + |
| 151_Ppyr_v3_TRINITY_DN17188_c0_g1_i1 | novel |  |
| 151_Ppyr_v3_TRINITY_DN12756_c0_g1_i1 | novel | + |
| 151_Ppyr_v3_TRINITY_DN888_c0_g2_i1 | novel | + |
| 151_Ppyr_v3_TRINITY_DN4568_c0_g1_i1 | novel |  |
| 151_Ppyr_v3_TRINITY_DN8548_c0_g3_i1 | novel |  |
| 151_Ppyr_v3_TRINITY_DN58520_c0_g1_i1 | novel |  |
| 151_Ppyr_v3_TRINITY_DN14365_c0_g1_i1 | novel | + |
| 151_Ppyr_v3_TRINITY_DN12318_c0_g1_i2 | novel | + |
| 151_Ppyr_v3_TRINITY_DN30894_c2_g1_i1 | novel |  |
| 151_Ppyr_v3_TRINITY_DN16302_c0_g1_i7 | novel |  |
| 151_Ppyr_v3_TRINITY_DN16302_c0_g1_i9 | novel |  |
| 151_Ppyr_v3_TRINITY_DN8691_c0_g1_i1 | novel | + |
| 151_Ppyr_v3_TRINITY_DN58074_c0_g1_i1 | novel |  |
| 151_Ppyr_v3_TRINITY_DN17778_c0_g1_i3 | novel | + |
| 151_Ppyr_v3_TRINITY_DN17778_c0_g1_i2 | novel |  |
| 151_Ppyr_v3_TRINITY_DN17778_c0_g1_i1 | novel |  |
| 151_Ppyr_v3_TRINITY_DN17778_c0_g2_i5 | novel |  |
| 151_Ppyr_v3_TRINITY_DN17778_c0_g2_i2 | novel |  |
| 151_Ppyr_v3_TRINITY_DN17778_c0_g2_i1 | novel |  |
| 151_Ppyr_v3_TRINITY_DN14800_c0_g1_i1 | novel | + |
| 151_Ppyr_v3_TRINITY_DN17889_c0_g1_i7 | novel |  |
| 151_Ppyr_v3_TRINITY_DN1208_c0_g1_i1 | novel |  |
| 151_Ppyr_v3_TRINITY_DN18057_c0_g1_i1 | novel |  |
| 151_Ppyr_v3_TRINITY_DN13010_c0_g1_i4 | novel | + |
| 151_Ppyr_v3_TRINITY_DN13010_c0_g1_i1 | novel | + |
| 151_Ppyr_v3_TRINITY_DN13010_c0_g1_i3 | novel | + |
| 151_Ppyr_v3_TRINITY_DN17799_c6_g3_i11 | novel |  |
| 151_Ppyr_v3_TRINITY_DN17799_c6_g3_i14 | novel |  |
| 151_Ppyr_v3_TRINITY_DN3035_c0_g1_i1 | novel |  |
| 151_Ppyr_v3_TRINITY_DN14034_c0_g1_i3 | novel |  |
| 151_Ppyr_v3_TRINITY_DN15764_c0_g1_i1 | novel | + |
| 151_Ppyr_v3_TRINITY_DN6755_c0_g1_i1 | novel | + |
| 151_Ppyr_v3_TRINITY_DN5991_c0_g1_i1 | novel |  |
| 151_Ppyr_v3_TRINITY_DN30884_c0_g1_i1 | novel |  |
| 151_Ppyr_v3_TRINITY_DN8174_c0_g1_i1 | novel | + |
| 151_Ppyr_v3_TRINITY_DN2338_c0_g1_i1 | novel |  |
| 151_Ppyr_v3_TRINITY_DN15444_c0_g3_i1 | novel |  |
| 151_Ppyr_v3_TRINITY_DN15444_c0_g2_i1 | novel |  |
| 151_Ppyr_v3_TRINITY_DN15444_c0_g1_i1 | novel |  |
| 151_Ppyr_v3_TRINITY_DN7008_c1_g1_i1 | novel | + |
| 151_Ppyr_v3_TRINITY_DN16723_c3_g1_i1 | novel | + |
| 151_Ppyr_v3_TRINITY_DN4571_c0_g1_i1 | novel |  |
| 151_Ppyr_v3_TRINITY_DN7697_c0_g1_i1 | novel | + |
| 151_Ppyr_v3_TRINITY_DN243_c0_g1_i1 | novel |  |
| 151_Ppyr_v3_TRINITY_DN14054_c0_g1_i1 | novel | + |
| 151_Ppyr_v3_TRINITY_DN16603_c1_g1_i1 | novel | + |
| 151_Ppyr_v3_TRINITY_DN4129_c0_g1_i1 | novel | + |
| 151_Ppyr_v3_TRINITY_DN11099_c0_g1_i1 | novel |  |
| 151_Ppyr_v3_TRINITY_DN6279_c0_g1_i1 | novel |  |
| 151_Ppyr_v3_TRINITY_DN16307_c0_g1_i1 | novel |  |
| 151_Ppyr_v3_TRINITY_DN4617_c0_g1_i1 | novel |  |
| 151_Ppyr_v3_TRINITY_DN8741_c0_g1_i1 | novel | + |
| 151_Ppyr_v3_TRINITY_DN15238_c0_g1_i1 | novel | + |
| 151_Ppyr_v3_TRINITY_DN17799_c6_g4_i2 | novel |  |
| 151_Ppyr_v3_TRINITY_DN15559_c1_g2_i1 | novel | + |
| 151_Ppyr_v3_TRINITY_DN16752_c1_g1_i1 | novel |  |
| 151_Ppyr_v3_TRINITY_DN40161_c0_g1_i1 | novel | + |
| 151_Ppyr_v3_TRINITY_DN17128_c1_g1_i1 | novel | + |
| 151_Ppyr_v3_TRINITY_DN51313_c0_g1_i1 | novel | + |
| 151_Ppyr_v3_TRINITY_DN6334_c0_g1_i1 | novel | + |
| 151_Ppyr_v3_TRINITY_DN6983_c0_g1_i1 | novel | + |
| 151_Ppyr_v3_TRINITY_DN16964_c1_g6_i1 | novel | + |
| 151_Ppyr_v3_TRINITY_DN12049_c0_g2_i1 | novel | + |
| 151_Ppyr_v3_TRINITY_DN15887_c0_g1_i1 | novel | + |
| 151_Ppyr_v3_TRINITY_DN11186_c0_g2_i1 | novel |  |
| 151_Ppyr_v3_TRINITY_DN15103_c0_g1_i1 | novel |  |
| 151_Ppyr_v3_TRINITY_DN16416_c0_g1_i10 | novel | + |
| 151_Ppyr_v3_TRINITY_DN9601_c0_g1_i1 | novel |  |
| 151_Ppyr_v3_TRINITY_DN1696_c0_g1_i1 | novel | + |
| 151_Ppyr_v3_TRINITY_DN12392_c0_g1_i1 | novel | + |
| 151_Ppyr_v3_TRINITY_DN11964_c0_g1_i1 | novel |  |
| 151_Ppyr_v3_TRINITY_DN17196_c0_g1_i1 | novel | + |
| 151_Ppyr_v3_TRINITY_DN6017_c0_g1_i1 | novel |  |
| 151_Ppyr_v3_TRINITY_DN10273_c0_g2_i1 | novel |  |
| 151_Ppyr_v3_TRINITY_DN6888_c0_g1_i1 | novel |  |
| 151_Ppyr_v3_TRINITY_DN7241_c0_g1_i1 | novel | + |
| 151_Ppyr_v3_TRINITY_DN10700_c0_g1_i1 | novel |  |
| 151_Ppyr_v3_TRINITY_DN10809_c0_g1_i1 | novel |  |
| 151_Ppyr_v3_TRINITY_DN15524_c0_g1_i5 | novel | + |
| 151_Ppyr_v3_TRINITY_DN17353_c0_g1_i2 | novel |  |
| 151_Ppyr_v3_TRINITY_DN41962_c0_g1_i1 | novel | + |
| 151_Ppyr_v3_TRINITY_DN16134_c1_g1_i1 | novel |  |
| 151_Ppyr_v3_TRINITY_DN16134_c1_g1_i2 | novel |  |
| 151_Ppyr_v3_TRINITY_DN17025_c0_g2_i2 | novel |  |
| 151_Ppyr_v3_TRINITY_DN17025_c0_g2_i1 | novel |  |
| 151_Ppyr_v3_TRINITY_DN14760_c1_g1_i1 | novel |  |
| 151_Ppyr_v3_TRINITY_DN42086_c0_g1_i1 | novel | + |
| 151_Ppyr_v3_TRINITY_DN58245_c0_g1_i1 | novel | + |
| 151_Ppyr_v3_TRINITY_DN6374_c0_g1_i1 | novel | + |
| 151_Ppyr_v3_TRINITY_DN12581_c0_g1_i2 | novel | + |
| 151_Ppyr_v3_TRINITY_DN14594_c0_g1_i1 | novel |  |
| 151_Ppyr_v3_TRINITY_DN13268_c1_g1_i1 | novel | + |
| 151_Ppyr_v3_TRINITY_DN7004_c0_g1_i1 | novel |  |
| 151_Ppyr_v3_TRINITY_DN11697_c1_g1_i1 | novel |  |
| 151_Ppyr_v3_TRINITY_DN11985_c0_g1_i1 | novel |  |
| 151_Ppyr_v3_TRINITY_DN2228_c0_g1_i1 | novel | + |
| 151_Ppyr_v3_TRINITY_DN14661_c0_g1_i1 | novel | + |
| 151_Ppyr_v3_TRINITY_DN15650_c1_g2_i1 | novel |  |
| 151_Ppyr_v3_TRINITY_DN8279_c0_g1_i1 | novel |  |
| 151_Ppyr_v3_TRINITY_DN23512_c0_g1_i1 | novel |  |
| 151_Ppyr_v3_TRINITY_DN5368_c0_g1_i1 | novel |  |
| 151_Ppyr_v3_TRINITY_DN19346_c0_g1_i1 | novel |  |
| 151_Ppyr_v3_TRINITY_DN17799_c6_g3_i7 | novel |  |
| 151_Ppyr_v3_TRINITY_DN16897_c1_g1_i1 | novel | + |
| 151_Ppyr_v3_TRINITY_DN9064_c1_g1_i1 | novel |  |
| 151_Ppyr_v3_TRINITY_DN12420_c0_g1_i1 | novel | + |
| 151_Ppyr_v3_TRINITY_DN16695_c0_g1_i1 | novel |  |
| 151_Ppyr_v3_TRINITY_DN5305_c0_g2_i1 | novel |  |
| 151_Ppyr_v3_TRINITY_DN3228_c0_g1_i1 | novel | + |
| 151_Ppyr_v3_TRINITY_DN2808_c0_g1_i1 | novel |  |
| 151_Ppyr_v3_TRINITY_DN17889_c0_g1_i1 | novel |  |
| 151_Ppyr_v3_TRINITY_DN9740_c0_g2_i1 | novel |  |
| 151_Ppyr_v3_TRINITY_DN9740_c0_g1_i2 | novel |  |
| 151_Ppyr_v3_TRINITY_DN17491_c1_g1_i2 | novel |  |
| 151_Ppyr_v3_TRINITY_DN17491_c0_g1_i4 | novel |  |
| 151_Ppyr_v3_TRINITY_DN17491_c1_g1_i3 | novel |  |
| 151_Ppyr_v3_TRINITY_DN11665_c0_g1_i1 | novel |  |
| 151_Ppyr_v3_TRINITY_DN8668_c1_g1_i1 | novel | + |
| 151_Ppyr_v3_TRINITY_DN8262_c0_g1_i1 | novel | + |
| 151_Ppyr_v3_TRINITY_DN50073_c0_g1_i1 | novel |  |
| 151_Ppyr_v3_TRINITY_DN10439_c0_g1_i1 | novel |  |
| 151_Ppyr_v3_TRINITY_DN5177_c0_g1_i1 | novel | + |
| 151_Ppyr_v3_TRINITY_DN2214_c0_g2_i1 | novel | + |
| 151_Ppyr_v3_TRINITY_DN2214_c0_g1_i1 | novel | + |
| 151_Ppyr_v3_TRINITY_DN15444_c0_g3_i1 | novel |  |
| 151_Ppyr_v3_TRINITY_DN5276_c0_g2_i1 | novel | + |
| 151_Ppyr_v3_TRINITY_DN2421_c0_g1_i1 | novel | + |
| 151_Ppyr_v3_TRINITY_DN11061_c0_g1_i1 | novel |  |
| 151_Ppyr_v3_TRINITY_DN111_c1_g1_i1 | novel |  |
| 151_Ppyr_v3_TRINITY_DN17024_c0_g1_i1 | novel | + |
| 151_Ppyr_v3_TRINITY_DN6688_c0_g1_i1 | novel | + |
| 151_Ppyr_v3_TRINITY_DN15103_c0_g2_i1 | novel |  |
| 151_Ppyr_v3_TRINITY_DN19775_c0_g1_i1 | novel | + |
| 151_Ppyr_v3_TRINITY_DN5227_c0_g1_i1 | novel |  |
| 151_Ppyr_v3_TRINITY_DN16243_c0_g1_i1 | novel | + |
| 151_Ppyr_v3_TRINITY_DN12416_c3_g1_i2 | novel | + |
| 151_Ppyr_v3_TRINITY_DN7933_c0_g1_i1 | novel |  |
| 151_Ppyr_v3_TRINITY_DN48878_c0_g1_i1 | novel | + |
| 151_Ppyr_v3_TRINITY_DN17517_c2_g1_i4 | novel |  |
| 151_Ppyr_v3_TRINITY_DN17517_c2_g1_i3 | novel |  |
| 151_Ppyr_v3_TRINITY_DN17761_c0_g1_i1 | novel |  |
| 151_Ppyr_v3_TRINITY_DN10825_c0_g1_i1 | novel |  |
| 151_Ppyr_v3_TRINITY_DN8701_c0_g1_i1 | novel | + |
| 151_Ppyr_v3_TRINITY_DN2953_c0_g2_i1 | novel | + |
| 151_Ppyr_v3_TRINITY_DN58007_c0_g1_i1 | novel | + |
| 151_Ppyr_v3_TRINITY_DN6616_c1_g1_i1 | novel | + |
| 151_Ppyr_v3_TRINITY_DN16768_c0_g2_i2 | novel | + |
| 151_Ppyr_v3_TRINITY_DN16768_c0_g2_i6 | novel | + |
| 151_Ppyr_v3_TRINITY_DN13875_c0_g1_i1 | novel |  |
| 151_Ppyr_v3_TRINITY_DN59691_c0_g1_i1 | novel |  |
| 151_Ppyr_v3_TRINITY_DN6937_c0_g1_i1 | novel | + |
| 151_Ppyr_v3_TRINITY_DN3566_c0_g1_i1 | novel | + |
| 151_Ppyr_v3_TRINITY_DN2343_c0_g1_i1 | novel | + |
| 151_Ppyr_v3_TRINITY_DN19646_c0_g1_i1 | novel |  |
| 151_Ppyr_v3_TRINITY_DN14255_c0_g1_i3 | novel | + |
| 151_Ppyr_v3_TRINITY_DN16243_c0_g3_i1 | novel | + |
| 151_Ppyr_v3_TRINITY_DN7824_c0_g1_i1 | novel | + |
| 151_Ppyr_v3_TRINITY_DN802_c0_g1_i1 | novel | + |
| 151_Ppyr_v3_TRINITY_DN1657_c0_g2_i1 | novel | + |
| 151_Ppyr_v3_TRINITY_DN15609_c0_g1_i1 | novel |  |
| 151_Ppyr_v3_TRINITY_DN2319_c1_g1_i1 | novel | + |
| 151_Ppyr_v3_TRINITY_DN11342_c0_g1_i1 | novel |  |
| 151_Ppyr_v3_TRINITY_DN47556_c0_g1_i1 | novel | + |
| 151_Ppyr_v3_TRINITY_DN16447_c0_g2_i1 | novel |  |
| 151_Ppyr_v3_TRINITY_DN15524_c0_g1_i2 | novel | + |
| 151_Ppyr_v3_TRINITY_DN11760_c0_g1_i1 | novel |  |
| 151_Ppyr_v3_TRINITY_DN1435_c3_g5_i1 | novel |  |
| 151_Ppyr_v3_TRINITY_DN1435_c3_g6_i1 | novel |  |
| 151_Ppyr_v3_TRINITY_DN1435_c3_g8_i1 | novel |  |
| 151_Ppyr_v3_TRINITY_DN5809_c1_g1_i1 | novel | + |
| 151_Ppyr_v3_TRINITY_DN5981_c0_g1_i1 | novel |  |
| 151_Ppyr_v3_TRINITY_DN24262_c0_g1_i1 | novel | + |
| 151_Ppyr_v3_TRINITY_DN6371_c0_g1_i1 | novel | + |
| 151_Ppyr_v3_TRINITY_DN2701_c0_g1_i1 | novel | + |
| 151_Ppyr_v3_TRINITY_DN20807_c0_g1_i1 | novel | + |
| 151_Ppyr_v3_TRINITY_DN16302_c0_g1_i6 | novel |  |
| 151_Ppyr_v3_TRINITY_DN16553_c0_g1_i1 | novel |  |
| 151_Ppyr_v3_TRINITY_DN27553_c0_g1_i1 | novel | + |
| 151_Ppyr_v3_TRINITY_DN7460_c0_g3_i1 | novel | + |
| 151_Ppyr_v3_TRINITY_DN17375_c0_g1_i1 | novel |  |
| 151_Ppyr_v3_TRINITY_DN5839_c0_g1_i1 | novel | + |
| 151_Ppyr_v3_TRINITY_DN16905_c0_g1_i1 | novel |  |
| 151_Ppyr_v3_TRINITY_DN49523_c0_g1_i1 | novel | + |
| 151_Ppyr_v3_TRINITY_DN6628_c0_g1_i1 | novel | + |
| 151_Ppyr_v3_TRINITY_DN3140_c0_g1_i1 | novel |  |
| 151_Ppyr_v3_TRINITY_DN13179_c0_g1_i3 | novel |  |
| 151_Ppyr_v3_TRINITY_DN13689_c0_g1_i1 | novel |  |
| 151_Ppyr_v3_TRINITY_DN17631_c0_g1_i2 | novel |  |
| 151_Ppyr_v3_TRINITY_DN14296_c0_g3_i1 | novel |  |
| 151_Ppyr_v3_TRINITY_DN13964_c0_g1_i1 | novel |  |
| 151_Ppyr_v3_TRINITY_DN52223_c0_g1_i1 | novel | + |
| 151_Ppyr_v3_TRINITY_DN31102_c0_g1_i1 | novel |  |
| 151_Ppyr_v3_TRINITY_DN11072_c0_g1_i1 | novel |  |
| 151_Ppyr_v3_TRINITY_DN12296_c0_g1_i1 | novel |  |
| 151_Ppyr_v3_TRINITY_DN41402_c0_g1_i1 | novel |  |
| 151_Ppyr_v3_TRINITY_DN40046_c0_g1_i1 | novel |  |
| 151_Ppyr_v3_TRINITY_DN23458_c0_g1_i1 | novel | + |
| 151_Ppyr_v3_TRINITY_DN43044_c0_g1_i1 | novel |  |
| 151_Ppyr_v3_TRINITY_DN13309_c0_g1_i5 | novel | + |
| 151_Ppyr_v3_TRINITY_DN11463_c0_g1_i1 | novel |  |

**Supplementary references:**

Chambers, M.C., Maclean, B., Burke, R., Amodei, D., Ruderman, D.L., Neumann, S., Gatto, L., Fischer, B., Pratt, B., Egertson, J., et al. (2012). A cross-platform toolkit for mass spectrometry and proteomics. *Nat Biotech* *30*, 918–920.

Goetz, M.A., Meinwald, J., and Eisner, T. (1981). Lucibufagins, IV. New defensive steroids and a pterin from the firefly *Photinus pyralis* (Coleoptera: Lampyridae). *Cellular and Molecular Life Sciences* *37*, 679–680.

Meinwald, J., Wiemer, D.F., and Eisner, T. (1979). Lucibufagins. 2. Esters of 12-oxo-2.beta.,5.beta.,11.alpha.-trihydroxybufalin, the major defensive steroids of the firefly *Photinus pyralis* (Coleoptera: Lampyridae). *J. Am. Chem. Soc*. *101*, 3055–3060.

Pluskal, T., Castillo, S., Villar-Briones, A., and Oresic, M. (2010). MZmine 2: modular framework for processing, visualizing, and analyzing mass spectrometry-based molecular profile data. *BMC Bioinformatics* *11*, 395.
